# Systemic immunostimulation induces glucocorticoid-mediated thymic involution succeeded by rebound hyperplasia which is impaired in aged recipients

**DOI:** 10.3389/fimmu.2024.1429912

**Published:** 2024-09-09

**Authors:** Craig P. Collins, Lam T. Khuat, Gail D. Sckisel, Logan V. Vick, Christine M. Minnar, Cordelia Dunai, Catherine T. Le, Brendan D. Curti, Marka Crittenden, Alexander Merleev, Michael Sheng, Nelson J. Chao, Emanual Maverakis, Spencer R. Rosario, Arta M. Monjazeb, Bruce R. Blazar, Dan L. Longo, Robert J. Canter, William J. Murphy

**Affiliations:** ^1^ Department of Dermatology, University of California, Davis, School of Medicine, Sacramento, CA, United States; ^2^ Earle A. Chiles Research Institute, Robert W. Franz Cancer Center, Providence Portland Medical Center, Portland, OR, United States; ^3^ Division of Hematologic Malignancies and Cellular Therapy, Department of Medicine, Duke University School of Medicine, Durham, NC, United States; ^4^ Biostatistics & Bioinformatics Department, Roswell Park, Roswell Comprehensive Cancer Center, Buffalo, NY, United States; ^5^ Department of Radiation Oncology, University of California, Davis Comprehensive Cancer Center, School of Medicine, Sacramento, CA, United States; ^6^ Department of Pediatrics, University of Minnesota, Minneapolis, MN, United States; ^7^ Department of Medicine, Harvard Medical School, Boston, MA, United States; ^8^ Division of Surgical Oncology, Department of Surgery, University of California, Davis Comprehensive Cancer Center, School of Medicine, Sacramento, CA, United States; ^9^ Department of Internal Medicine, Division of Hematology and Oncology, University of California, Davis, School of Medicine, Sacramento, CA, United States

**Keywords:** immune therapy, thymic involution, age, viral infection, stress, glucocoricoids

## Abstract

The thymus is the central organ involved with T-cell development and the production of naïve T cells. During normal aging, the thymus undergoes marked involution, reducing naïve T-cell output and resulting in a predominance of long-lived memory T cells in the periphery. Outside of aging, systemic stress responses that induce corticosteroids (CS), or other insults such as radiation exposure, induce thymocyte apoptosis, resulting in a transient acute thymic involution with subsequent recovery occurring after cessation of the stimulus. Despite the increasing utilization of immunostimulatory regimens in cancer, effects on the thymus and naïve T cell output have not been well characterized. Using both mouse and human systems, the thymic effects of systemic immunostimulatory regimens, such as high dose IL-2 (HD IL-2) with or without agonistic anti-CD40 mAbs and acute primary viral infection, were investigated. These regimens produced a marked acute thymic involution in mice, which correlated with elevated serum glucocorticoid levels and a diminishment of naïve T cells in the periphery. This effect was transient and followed with a rapid thymic “rebound” effect, in which an even greater quantity of thymocytes was observed compared to controls. Similar results were observed in humans, as patients receiving HD IL-2 treatment for cancer demonstrated significantly increased cortisol levels, accompanied by decreased peripheral blood naïve T cells and reduced T-cell receptor excision circles (TRECs), a marker indicative of recent thymic emigrants. Mice adrenalectomized prior to receiving immunotherapy or viral infection demonstrated protection from this glucocorticoid-mediated thymic involution, despite experiencing a substantially higher inflammatory cytokine response and increased immunopathology. Investigation into the effects of immunostimulation on middle aged (7-12 months) and advance aged (22-24 months) mice, which had already undergone significant thymic involution and had a diminished naïve T cell population in the periphery at baseline, revealed that even further involution was incurred. Thymic rebound hyperplasia, however, only occurred in young and middle-aged recipients, while advance aged not only lacked this rebound hyperplasia, but were entirely absent of any indication of thymic restoration. This coincided with prolonged deficits in naïve T cell numbers in advanced aged recipients, further skewing the already memory dominant T cell pool. These results demonstrate that, in both mice and humans, systemic immunostimulatory cancer therapies, as well as immune challenges like subacute viral infections, have the potential to induce profound, but transient, glucocorticoid-mediated thymic involution and substantially reduced thymic output, resulting in the reduction of peripheral naive T cells. This can then be followed by a marked rebound effect with naïve T cell restoration, events that were shown not to occur in advanced-aged mice.

## Introduction

The thymus is the central organ for T-cell development and differentiation. CD4/CD8 double-positive (DP) T-cell progenitors, which comprise the majority of thymocytes within the thymus, undergo thymic education, with mature single positive CD4 or CD8 naïve T cells exiting the thymus into the periphery, comprising the naïve T-cell pool (1), while those that fail the process are eliminated. However, soon after adulthood, the mammalian thymus undergoes a continuous process of thymic involution, gradually causing a marked reduction of naïve T-cell output (2–4), as well as a skewing of the overall T-cell population towards a memory phenotype. Thymic involution can also occur due to DP apoptosis mediated by stimuli outside of normal education, such as radiation (5–7), exposure to pro-inflammatory cytokines such as tumor necrosis factor (TNF) (8), as well as being induced by glucocorticoids (9–11) (cortisol and corticosterone (CS), the principal glucocorticoids in humans and in rodents, respectively). Interestingly, it has been reported that after cessation of radiation therapy or chemotherapy, a thymic rebound hyperplasia can occur, resulting in higher levels of thymic cellularity in comparison to homeostatic baselines (12, 13). This phenomenon has not been shown in the elderly, however, with reports of thymic rebound hyperplasia excluding those above the age of 60 (14). Thymic function and naïve T cell output naturally diminishes with age, but in other conditions that incur T-cell deficiency, such as that observed with HIV infection (15, 16), or after hematopoietic stem cell transplantation (17, 18), the output of naïve T cells, which are evidence of thymic function, can still be observed in humans. This has been extensively demonstrated by quantification of T-cell receptor excision circles (TRECs) generated during initial TCR rearrangement in the thymus, which, due to not being replicated after this initial rearrangement, are now regarded as indicators of recent thymic emigrants (19).

Immunotherapy (IT) is being increasingly incorporated into many cancer treatment regimens. The systemic use of immunostimulatory regimens involving cytokines, immunomodulatory antibodies, oncolytic viruses, and adoptive cellular immunotherapies such as chimeric antigen receptor (CAR) T cells, have become frontline options in cancer treatments, often being combined with conventional cytoreductive conditioning and molecular targeted regimens. Despite this increasing application, however, the effects of systemic immunostimulatory regimens on thymic function, naive T-cell production, and on stress responses have not been well characterized. In the studies presented here, multiple models of systemic immune stimulation with systemic immunotherapies, as well as subacute viral infection challenges, were utilized to determine the impact on the thymus and naïve T-cell content, which could be of clinical interest when considering the use of glucocorticoids to protect against these effects.

## Materials and methods

### Mice and adrenalectomy

7-12 week old wild-type C57BL/6 and BALB/c mice were purchased from Taconic Biosciences (Germantown, NY, USA) or The Jackson Laboratory (Bar Harbor, ME, USA). Mice for aged studies were purchased from The Jackson Lab at 7-12 weeks of age and aged out to 7-12 months old (middle aged) or 22+ months old in the SPF vivarium of the Institute of Regenerative Cures (University of California, Davis). SHAM and adrenalectomized C57BL/6 and BALB/c mice were obtained from Jackson Laboratories (Bar Harbor, ME, USA) (20). Briefly, the adrenalectomies were performed at The Jackson Laboratory at approximately two months of age, and mice were shipped 1-2 weeks after surgery. Mice were then allowed a full two weeks after being received to recover, while on a specialized saline treatment. SHAM mice were provided water *ad libitum*, while adrenalectomized mice were maintained on 1% saline as recommended by The Jackson Laboratory. All mice used in studies were female. All mice were maintained at the UC Davis Medical Center’s vivarium in accordance with IACUC standards.

### Reagents

The agonistic anti-mouse CD40 antibody (clone FGK115B3) was generated via ascites production in our laboratory as previously described (21). The endotoxin level of the anti-CD40 was less than one endotoxin unit/mg antibody as determined by a quantitative chromogenic limulus amebocyte lysate kit (Bio Whittaker, Lonza, Switzerland). Recombinant human interleukin-2 (rhIL-2; TECIN Teceleukin, Roche) was provided by the National Cancer Institute (NCI, Frederick, MD, USA). Some mice received rat IgG (Jackson ImmunoResearch Laboratories Inc., PA, USA) as a control for anti-CD40 administration.

### Murine cytomegalovirus infection

The MCMV Smith strain was obtained from American Type Culture Collection (Manassas, VA, VR-1399) and maintained by repeated salivary gland passage through BALB/c mice. MCMV was tittered via a plaque forming unit (PFU) assay, as published (22). MCMV (1x10^4^ PFU, a subacute dose of infection) was administered i.p. in 0.2 mL of RPMI (Gibco Laboratories, Grand Island, NY) or at 1x10^5^ PFU (LD^50 for advance aged mice), while uninfected controls received 0.2 mL of RPMI (also via i.p. injection) only. Mice were monitored for signs of stress throughout the experiment and euthanized if moribund, or upon losing 20% body weight.

### Tumor studies

*In Vivo Immunotherapeutic Treatment Regimens (anti-CD40/rhIL-2 and anti-PD-1)* Agonistic anti-CD40 and rhIL-2 was administered in mice as previously described (23). Briefly, agonistic anti-CD40 or rat IgG (Jackson Immunoresearch Laboratories Inc., PA, USA) was administered at 65µg/0.2mL PBS per dose in BALB/c mice, or 80µg/0.2mL PBS per dose in C57BL/6 mice daily for 5 days (days 0-4). rhIL-2 or PBS alone was administered at 10^6^ IU/0.2mL PBS per dose for BALB/c mice or 2x10^6^ IU/0.2mL per dose for C57BL/6 mice twice per week for 2 weeks (days 1, 4, 8, 11).

Anti-PD-1 (clone 29F.1A12, catalog #BE0273, Bioxcell, NH, USA) was administered intraperitoneally at 500ug in 0.2 mL PBS at days 0 and 6, and then 250ug in 0.2mL PBS at day 8 and every 2 days afterwards until mice were harvested. Control mice were given 500ug hIgG in 0.2mL PBS on days 0 and 6, and then 250ug in 0.2mL PBS at day 8 and every 2 days afterwards until mice were harvested. Tumor measurements were taken on the same days that mice were given treatment.

### Clinical HD IL-2 trial and measurement of single joint T-cell receptor excision circles

Blood samples were obtained from patients with metastatic melanoma (NCT 01416831, as approved by the Providence Cancer Center IRB, located in Portland, Oregon) enrolled in Phase II high dose (HD) IL-2 trials. Patients received HD IL-2 [Proleukin (aldesleukin)] at 6 x 10^5^ IU/kg of body weight by intravenous injection every eight hours for 14 doses. Samples were obtained from patients at days 0, 2, 8, and 28.

CD3^+^ cells from patients samples were isolated from PBMCs using MACS magnetic microbeads (catalog no. 130-097-043, Miltenyi-Biotech, Bergisch Gladbach, North Rhine-Westphalia, Germany) and signal sjTRECs were then quantified as described elsewhere (24). Briefly, cells were lysed in 100 μg/mL proteinase K (catalog no. 3115828001, Roche, Basel, Switzerland) at 10^7^ cells/mL. A CFX96 thermocycler (Bio-Rad) was used to perform real time quantitative-PCR on 5 μL of lysate using Platinum Taq (catalog no. 15966005, ThermoFisher Scientific, Waltham, MA, USA) with the primers cacatccctttcaaccatgct and gccagctgcagggtttagg plus probe FAM-acacctctg-ZEN-gtttttgtaaaggtgcccact-3IABkFQ (Integrated DNA Technologies, Coralville, IA, USA). A standard curve was generated by performing RT q-PCR on known quantitates of human sjTRECs plasmids, and then CFX Manager software (Bio-Rad, Hercules, CA, USA) was used to determine the number of sjTRECs per 10^5^ cells.

### Cell preparation, antibodies, and flow cytometry analysis

Thymuses were mechanically dissected. In brief, thymuses were harvested from animals and placed in PBS, being processed via grinding between the frosted side of two frosted microscope slides (Fisher Scientific, 22-037-246). Spleens were similarly placed in PBS after extraction, and grinded in a culture plate well with the plunger of a 3mL syringe. Spleens and thymuses were then filtered through a 100 micron filter (Corning, 431752). Afterwards, both spleens and thymuses were treated with red blood cell (RBC) lysis buffer (Biolegend, 420302) for 5 minutes and removed afterwards, according to the manufacturer’s protocol. Cells were then counted using a Coulter Counter (Beckman Coulter Life Sciences, Z1 Series Coulter Counter). Single cell suspensions were then standardized to 1 million cells per 50uL of staining buffer (25).

Single-cell suspensions were plated at 1 million cells and stained with a viability dye (Zombie NIR) for 20 minutes. Afterwards, they were incubated with CD16/CD32 Fc Block (catalog no. 553141, BD Pharmingen, San Diego, CA, USA) for 10 minutes, and then co-incubated with surface antibodies for 20 minutes at 4°C, followed by washing with staining buffer (phosphate-buffered saline + 1% fetal bovine serum). Flow cytometry analysis was performed with a LSR Fortessa cell analyzer (BD Biosciences, San Jose, CA, USA), and data was analyzed using FlowJo v10 software (FlowJo, Ashland, OR, USA). The following mouse and human fluorochrome-conjugated monoclonal antibodies were used, having been purchased from BioLegend (San Diego, CA, USA): CD44–Pacific Blue (IM7), CD3-BV785 (17A2), CD4-BV711 (RM4-5), CD8-BV605 (53-6.7), CD62L-PE-Cy7 (MEL-14), CD45RA-BV421 (H100), CD45RO-PE-Cy7 (UCHL1), and HLA-DR-PerCP-Cy5.5 (L243).

For annexin V and propidium iodide (PI) staining, single-cell suspensions (1×10^6^ to 5×10^6^ cells/mL) were stained with Annexin V–Pacific Blue (catalog no. 640918, BioLegend, San Diego, CA, USA). Cells were then incubated in the dark for 15 minutes and stained with PI (catalog no. P3556, Life Technologies, Carlsbad, CA, USA) following the manufacturer’s protocol and were analyzed by flow cytometry within 10 minutes.

### Mouse serum corticosterone, human cortisol, and mouse cytokine assessment

Mouse blood samples were collected at various timepoints by tail vein bleeding. Serum samples were obtained by centrifugation of blood samples at 10000 rpm for 10 minutes. Corticosterone in the serum was then measured by a corticosterone ELISA kit (catalog no. KGE009, R&D Systems, MN, USA) per the manufacturer’s instructions.

Patient samples were provided at the timepoints described in the HD IL-2 trial section. Serum was collected by centrifugation of blood samples at 10000 RPM for 10 minutes. Serum samples were assayed via Enzo Human Cortisol ELISA kit per the manufacturer’s instructions (catalog no. ADI-900-097, Enzo Life Sciences, Farmingdale, NY, USA).

Mouse serum cytokines were measured by cytometric bead array flex set kits (BD Biosciences, San Jose, CA, USA): mouse TNF (catalog no. 558299), mouse IL-6 (catalog no. 558301), mouse IFN-γ (catalog no. 558296), and mouse IL-1β (catalog no. 560232). Serum samples were diluted 1:4 using the assay diluent solution provided in the kit. Capture beads and detection beads were added as described in the manufacturer’s protocol. Cytokine concentration was measured by flow cytometry with the LSR Fortessa cell analyzer (BD Biosciences, San Jose, CA, USA).

### RNA sequencing

Messenger RNA (mRNA) was isolated from total RNA using poly-T oligo-attached magnetic beads. This RNA was subsequently fragmented, and the first strand of complementary DNA (cDNA) was synthesized using random hexamer primers. This was followed by second-strand cDNA synthesis. The resulting library was evaluated using Qubit for quantification and real-time PCR, and a bioanalyzer was used for size distribution detection. Quantified libraries were then pooled and sequenced using a 2 × 150 bp paired-end method on Illumina NovaSeq 6000 sequencer. Further information can be found at the linked reference.

### RNAseq data analysis

The raw reads in fastq format were aligned to the mm10 reference mouse genome using Hisat2 (v2.0.5) [x1]. FeatureCounts (v1.5.0-p3) [x2] was employed to count the number of reads mapping to each gene. Differential gene expression analysis was carried out using the DESeq2 R package (v1.40.2) [23]. The P-values obtained from this analysis were adjusted using the Benjamini and Hochberg’s approach to control the false discovery rate. Genes with an adjusted P-value <=0.05 and a fold change >=2 were identified as differentially expressed. The “fgsea” R package (v.1.26.0) [24] was employed to conduct gene set enrichment analysis and to visualize the enriched gene sets. A clustered heatmap was generated using the regularized log-transformed data with the help of the “pheatmap” R package (v.1.0.12).

### Data analysis and statistics

Statistical analysis was performed using GraphPad Prism v6.02 (GraphPad Software Inc., CA, USA). Data were expressed as mean ± standard error of the mean (SEM). A non-parametric Mann-Whitney U-test was used to compare two unpaired groups. For analysis of three or more groups, the non-parametric ANOVA was performed with a Bonferroni post-test. An adjusted *p* value of <0.05 was considered significant. Experiments were performed 1-4 times, as further specified in figure legends. Mouse numbers per harvest ranged between 1-6 mice, with numbers shown in figures representing combined harvests through repeated experiments.

## Results

### Systemic immunostimulatory regimens result in rapid acute thymic involution in mice followed by thymic hyperplasia rebound in mice

**Figure 1:**
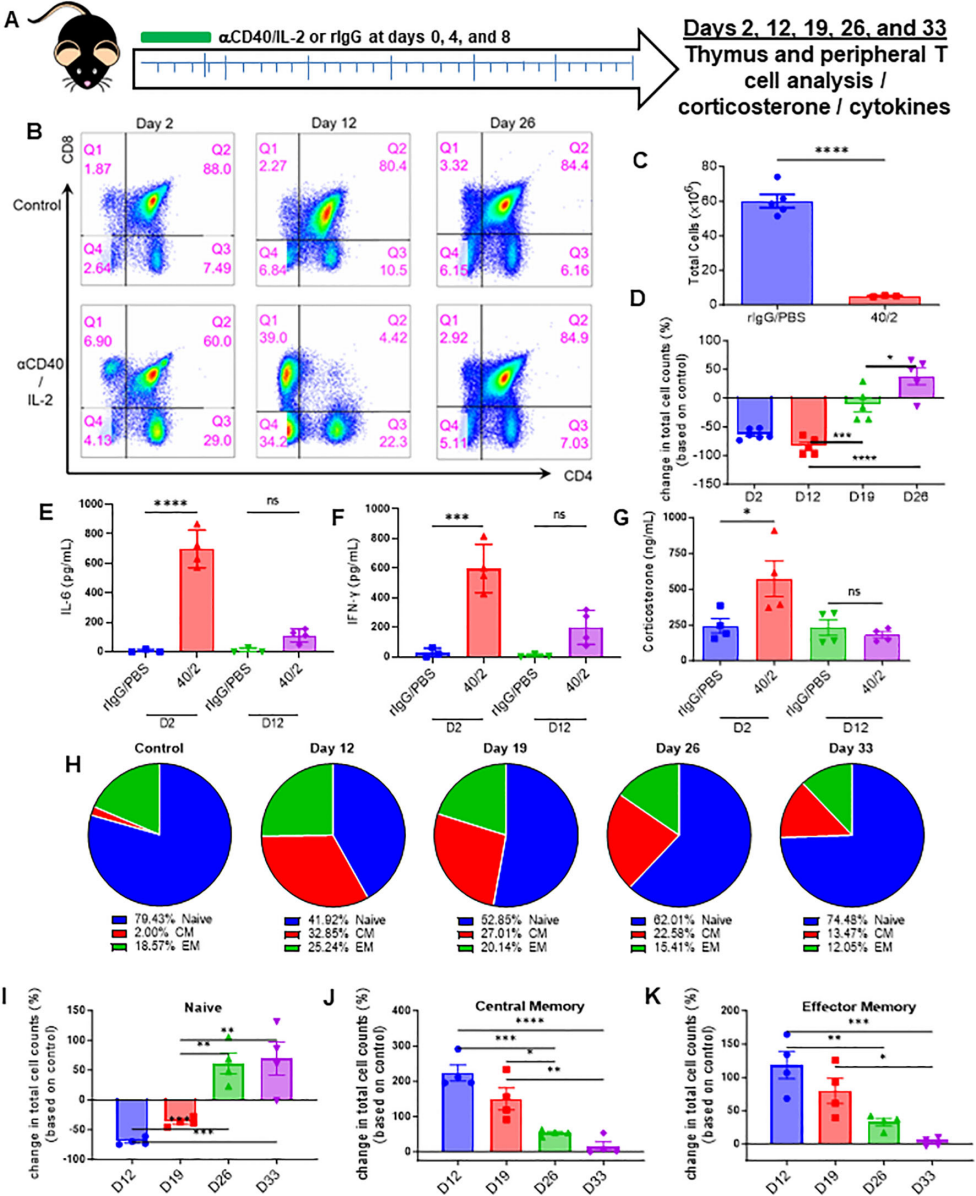
Strong systemic immunostimulatory regimens result in rapid acute thymic involution in mice. **(A)** Schema – C57BL/6 mice were given either anti-CD40 (aCD40, 65μg) in combination with rhIL-2 (5x10^6^ IU) (aCD40/IL-2 group) or rat IgG (control group) intraperitoneally on days 0, 4, and 8. Mice were monitored over the course of 33 days, with takedowns occurring at days 2, 12, 19, 26, and 33. **(B)** Representative flow cytometry staining of CD4^+^ vs CD8^+^ (previously gated on CD3^+^) thymocytes from days 2, 12, and 26. **(C)** Thymic cellularity of mice on day 2 post injection. **(D)** Fold change of double positive thymocytes in mice receiving aCD40/IL-2 in comparison to control mice at days 2, 12, 19, and 26. **(E, F)**. Serum levels of IL-6 and IFN-y at day 2 and day 12. **(G)** Serum corticosterone levels of mice at days 2 and 12 post injection. **(H)** Percentages of peripheral naïve (CD44^-^CD62L^+^ and CD44^-^CD62L^-^), central memory (CM) (CD44^+^CD62L^+^), or effector memory (EM) (CD44^+^CD62L^-^) populations of CD3^+^ over the course of the 33-day experiment. **(I–K)** Percentage change in peripheral total naïve, central memory, and effector memory CD3^+^ cell counts when compared to baseline controls over the course of the 33-day experiment **(D–K)** SEM bars, n=3-5 mice per group, representative of 2 experiments. One-way ANOVA with multiple comparisons based on means between groups was used to determine statistical significance; P<0.05*, P<0.01**, P<0.001***, P<0.0001****. **(C)** SEM bars, n=3-5 mice per group, representative of 2 experiments. Student’s *t* test was used to determine statistical significance; P<0.05*, P<0.01**, P<0.001***, P<0.0001****. ns, non-significant.

Immunostimulatory regimens involving systemic administration of cytokines such as IL-2, IL-15, IL-12, as well as TLR agonists and immunostimulatory antibodies, have been increasingly applied clinically in multiple cancers (26, 27). However, the primary focus of such regimens has been the production of immune mediated anti-tumor effects, while assessment of other aspects of immune cell development and organs critical for immune cell development, such as the thymus, have been largely uncharacterized. The effects of HD IL-2 on the thymus were first assessed *in vivo*, given its relevance as a cancer immune therapy (28–30). Mice were treated with IL-2 in combination with agonistic antibodies to CD40, a combination that was previously demonstrated to result in marked synergistic anti-tumor effects (21, 31, 32), to determine how the thymus was effected during and after administration (**Figure 1A**). Flow cytometric analysis of the thymus over the course of 33 days (**Figure 1A**, schema) revealed an almost complete loss of the CD4/CD8 double-positive population as early as 2 days after treatment (**Figure 1B**, days 2 and 12, **Supplementary Figures 1A, B**). In conjunction with the loss of this population, a striking decrease in total thymic cellularity was observed (**Figure 1C**). Interestingly, after cessation of the treatment, a thymic recovery occurred and a significant rebound effect was observed within this double positive population, as demonstrated by a gradual restoration of the population which notably surpassed that observed in the control group (**Figure 1D**, Days 19 and 26).

The effects of αCD40/IL-2 treatment on serum cytokines and inflammatory factors were next assessed. Significantly elevated systemic levels of pro-inflammatory cytokines IL-6 and IFN-γ were observed at day 2 (**Figures 1E, F**), indicative of strong systemic immunostimulation. Interestingly, serum CS levels were also markedly elevated with immunotherapy treatment. All values returned to baseline levels when treatments ended, which was when thymic reconstitution was observed (**Figure 1G**, day 12, four days after final treatment). Assessment on the effects on peripheral T cells, notably naïve T- cells, indicated this thymic involution was followed with a significant contraction of naïve T cells and an elevation of memory T-cell percentages. Thymocyte recovery was then also followed with a restoration of the naïve T-cell population and a receding of the memory population (**Figures 1H–K**; **Supplementary Figure 1C**).

These *in vivo* results demonstrated that systemic immunostimulatory regimens capable of activating peripheral T cells to produce anti-tumor effects, could also induce stress responses as indicated by elevated CS levels. These stress responses also corresponded with a profound, though transient, thymic involution, which was then followed by a gradual rebound after cessation of therapy, resulting in not only greater thymocyte cellularity, but also a restoration of peripheral naïve T cells.

### Sub-acute MCMV infection induces thymic involution and a corticosterone response in absence of elevated pro-inflammatory cytokines

HD IL-2 and anti-CD40/IL-2 induced marked pro-inflammatory cytokine and CS responses. While glucocorticoids have been found to be a driver of apoptosis in T cells (33), TNF has similarly been demonstrated to contribute to thymic progenitor apoptosis (34, 35), necessitating models in which these responses could be incurred independently from each other to implicate a primary driver of thymic involution or apoptosis.

Primary acute MCMV infection in mice has been demonstrated to induce CS responses in mice (36). Acute primary MCMV infection also induces robust proinflammatory cytokine responses, but by lowering the viral challenge, a sub-acute infection with minimal systemic pro-inflammatory response can be produced (**Supplementary Figure 2A**). Using this sub-acute MCMV infection model, it was observed that thymic involution still occurred (**Supplementary Figure 2B**), in conjunction with a marked CS response (**Supplementary Figure 2C**), as well as a transition from the naïve dominant T-cell pool becoming memory dominant (**Supplementary Figures 2D–G**). The CS response was notably transient and preceded thymic involution; the CS peak observed at 36 hours post-infection had subsided by day 2, while thymic involution did not occur during this peak, but rather at day 4 (**Supplementary Figures 2B, C, H**). This involution occurred in absence of a notable proinflammatory cytokine response, as measured by serum levels of TNF and IL-6, as well as the genes that transcribed these cytokines (**Supplementary Figures 2I–L**). These data indicate that a strong pro-inflammatory cytokine response does not act as the primary driver of thymic involution during immune stimulation or challenge, but rather, that it is a phenomenon instigated by systemic CS.

### High-dose IL-2 therapy results in significant decrease in thymic output, concurrent T-cell activation, and increased glucocorticoids in both mice and humans

**Figure 2:**
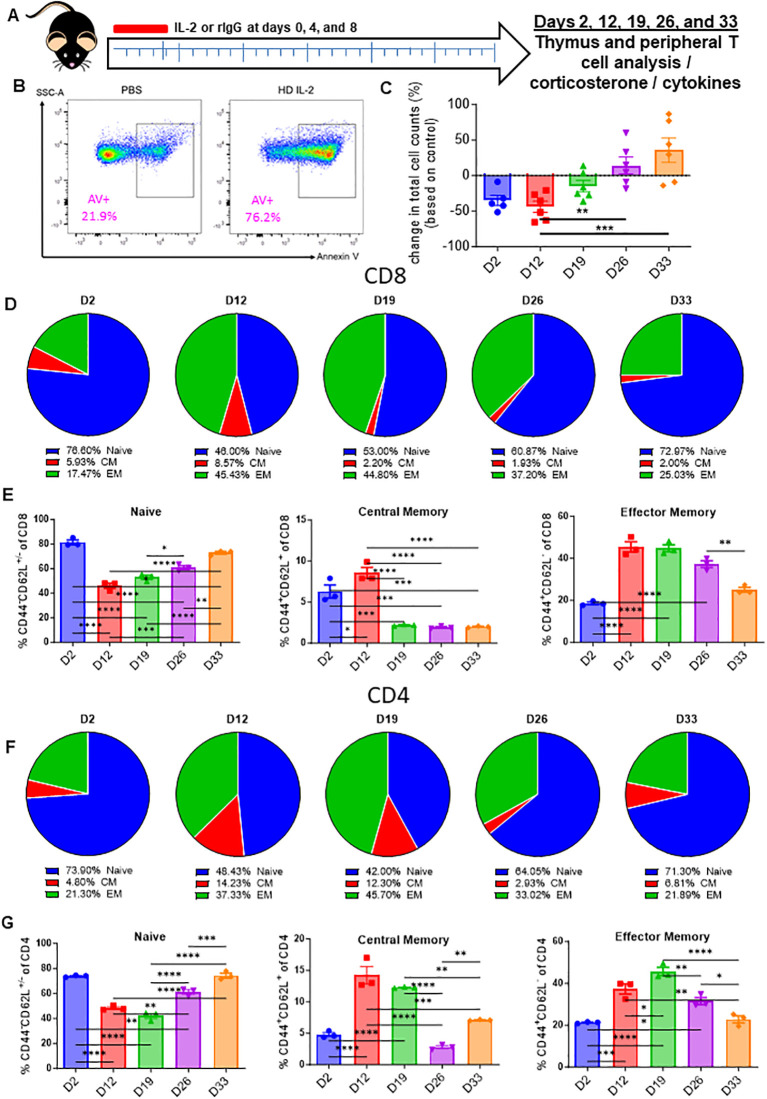
High-dose IL-2 induces apoptosis driven thymic involution. **(A)** Schema – Mice were administered 5x10^6^ IU of IL-2 or rat IgG at days 0, 4, and 8 intraperitoneally. Mice were monitored over the course of 33 days. Mice were taken down at days 2, 12, 19, 26, and 33. **(B)** Annexin V representative flow staining on thymuses from mice 24 hours after IL-2 administration **(C)** Thymic cellularity fold change of HD IL-2 treated mice vs controls over the course of the experiments. **(D, F)** Pie chart breakdowns of the percentages of naïve, central memory, and effector memory populations in CD8^+^ **(D)** and CD4^+^ **(F)** T cell subsets over the course of the experiments. **(E, G)** Percentages of naïve, effector memory, and central memory populations in CD8^+^ **(E)** and CD4^+^ **(G)** T cell subsets over the course of the experiments. **(C, E, G)** SEM bars, n=3-6 mice per group, representative of 1-2 experiments. One-way ANOVA with multiple comparisons based on means between groups was used to determine statistical significance; P<0.05*, P<0.01**, P<0.001***, P<0.0001****.

HD IL-2 is FDA-approved for the treatment of renal cell carcinoma and other cancers (26). To model this preclinically, HD IL-2 was administered in mice, which were then assessed for thymic output in a time course study (**Figure 2A**). Flow analysis of the thymus revealed apoptosis in CD3 T-cells was markedly elevated in IL-2 treated mice as early as 24 hours after injection (**Figure 2B**; **Supplementary Figure 4A**). In line with what was observed during treatment with anti-CD40/IL-2, HD IL-2 induced a significant loss of thymocytes immediately after the first dose (**Figure 2C**; **Supplementary Figure 3B**) which correlated to increased levels of CD3^+^ T cell apoptosis (**Supplementary Figure 3B**). Mirroring previous data, this was proven to be transient, with a rebound effect being observed after cessation of treatment (**Figure 2C**; **Supplementary Figure 3C**), in conjunction with serum corticosterone levels peaking at day 2 and significantly reducing over time after the challenge, also mirroring previous data (**Supplementary Figure 3D**). While alterations were observable in single positive CD4 and CD8 and double negative thymocytes, it was the double positive thymocyte population which showed the largest differences over the course of the experiment, again mirroring previous data showing a loss and gradual rebound effect (**Supplementary Figure 3E**). A shift of the peripheral T-cell naïve to memory ratio was observed over the course of the treatment, initially being a mostly naïve dominant phenotype and gradually transitioning to one that saw a demonstrable growth of the memory population, though this effect seemed to be in the process of reversing back to homeostatic conditions at the end of the observed period (**Figures 2D–G**; **Supplementary Figures 3F, G**). This was observed in both CD8 (**Figures 2D, E**; **Supplementary Figure 3F**) and CD4 (**Figures 2F, G**; **Supplementary Figure 3G**) subsets of T-cells. Regardless of subset and despite not overcoming naïve T cells in raw percentages, memory fold expansion during treatment eclipsed that which was observed in the naïve pool, which saw a fold decrease in comparison to controls (**Figures 2E, G**).

**Figure 3:**
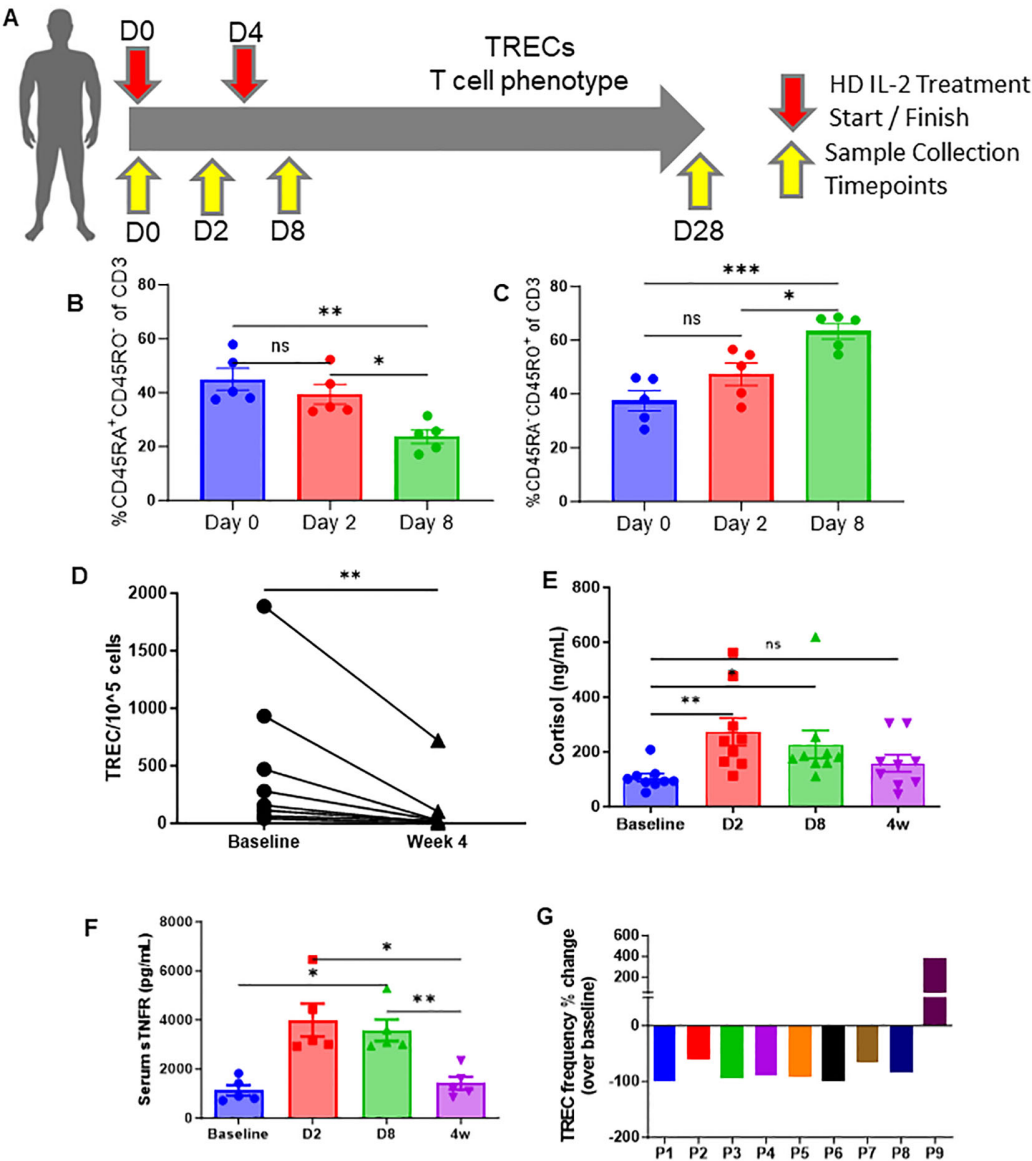
High-dose (HD) IL-2 therapy results in significant decrease in thymic output, concurrent T-cell activation, and increased glucocorticoids humans. **(A)** Schema – patients with metastatic melanoma or renal cell carcinoma received 6x10^5^ IU of IL-2 every 8 hours for 14 doses. Blood samples were collected from patients at days 0, 2, 8, and 28. **(B, C)** Percentage of naïve (CD45RA^+^CD45RO^+^, 6H) and memory (CD45RA^-^CD45RO^+^, 6I) of CD3^+^ T cells from peripheral blood of patients at days 0, 2, and 8. **(D)** Quantification of TREC^+^ cell numbers in the peripheral blood of patients at baseline vs week 4. **(E, F)** Serum cortisol and sTNFR levels of patients at days 0, 2, 8, and 28 days. **(G)**: TREC frequency fold change of individual patients at day 28 when compared to homeostatic baselines before treatment. **(B, C, E, F)**: SEM bars, n=5-9 patients per group, representative of samples from 1 clinical trial. One-way ANOVA with multiple comparisons based on means between groups was used to determine statistical significance in **(B, C)** while samples were paired for ANOVA analysis across baseline, D2, D8, and 4w in **(F, G)**; P<0.05*, P<0.01**, P<0.001***. **(D)**: Paired *t* test was used to determine statistical significance; P<0.05*. ns, non-significant.

To determine if the observed effects of HD IL-2 on glucocorticoid-mediated mouse thymic output and naïve T cells also occurred clinically, PBMCs were obtained from consenting cancer patients undergoing HD IL-2 therapy both before treatment and throughout the course of 1-4 weeks after, which were used to perform T-cell phenotyping (**Figure 3A**). In these studies, sjTRECs were assessed as an indicator of recent thymic emigrants, a metric which has been published on extensively (19, 24). In accordance with the mouse data, patients undergoing HD IL-2 therapy demonstrated a marked reduction in naïve T cells (CD45RA^+^ CD45RO^-^), and an inflation of the memory population (CD45RA^-^CD45RO^+^), which was evident as early as day 2 and became more pronounced by day 8 (**Figures 3B, C**; **Supplementary Figures 4A, B**), which was also accompanied by higher levels of activation as determined via HLA-DR (**Supplementary Figure 4C**). Following HD IL-2 treatments, in both studies, CD8 sjTREC levels were significantly lower by day 8, while sjTREC levels were lower in CD3 T cells 4 weeks after treatment, as well as the CD4 and CD8 subsets, correlating with the reduction of naïve T cells (**Figure 3D**, **Supplementary Figures 4D, E**), being indicative of reduced thymic output. It is also possible that memory T cell expansion is involved in the decreased levels of sjTRECs seen at day 8, reminiscent of mouse data shown earlier in the manuscript. Mirroring the transient peak in corticosterone preceding thymic apoptosis and involution that was observed in the HD IL-2 mouse studies, increased cortisol and pro-inflammatory sTNFR levels were observed in the patients during therapy which then subsided after treatment, coinciding with lower sjTREC levels being observed (**Figures 3E–G**). Humans were not examined at later time points to assess the rebound of naïve T cells. These results of these human studies, in tandem with the data produced by *in vivo* mouse studies, strongly suggest that systemic immunostimulatory regimens and immune challenges, such as HD IL-2, TLR engagement, and viral infection and other pathogen stimuli, induce corticosteroid responses that correlate with decreased sjTREC positive T cells in the periphery, specifically in CD8 T cells at the day 8 timepoint, likely due to inhibitory effects on the thymus through glucocorticoid-mediated involution.

### Adrenalectomy blunts the CS response and can protect the thymus from immunotherapy or viral infection-associated thymic involution

**Figure 4:**
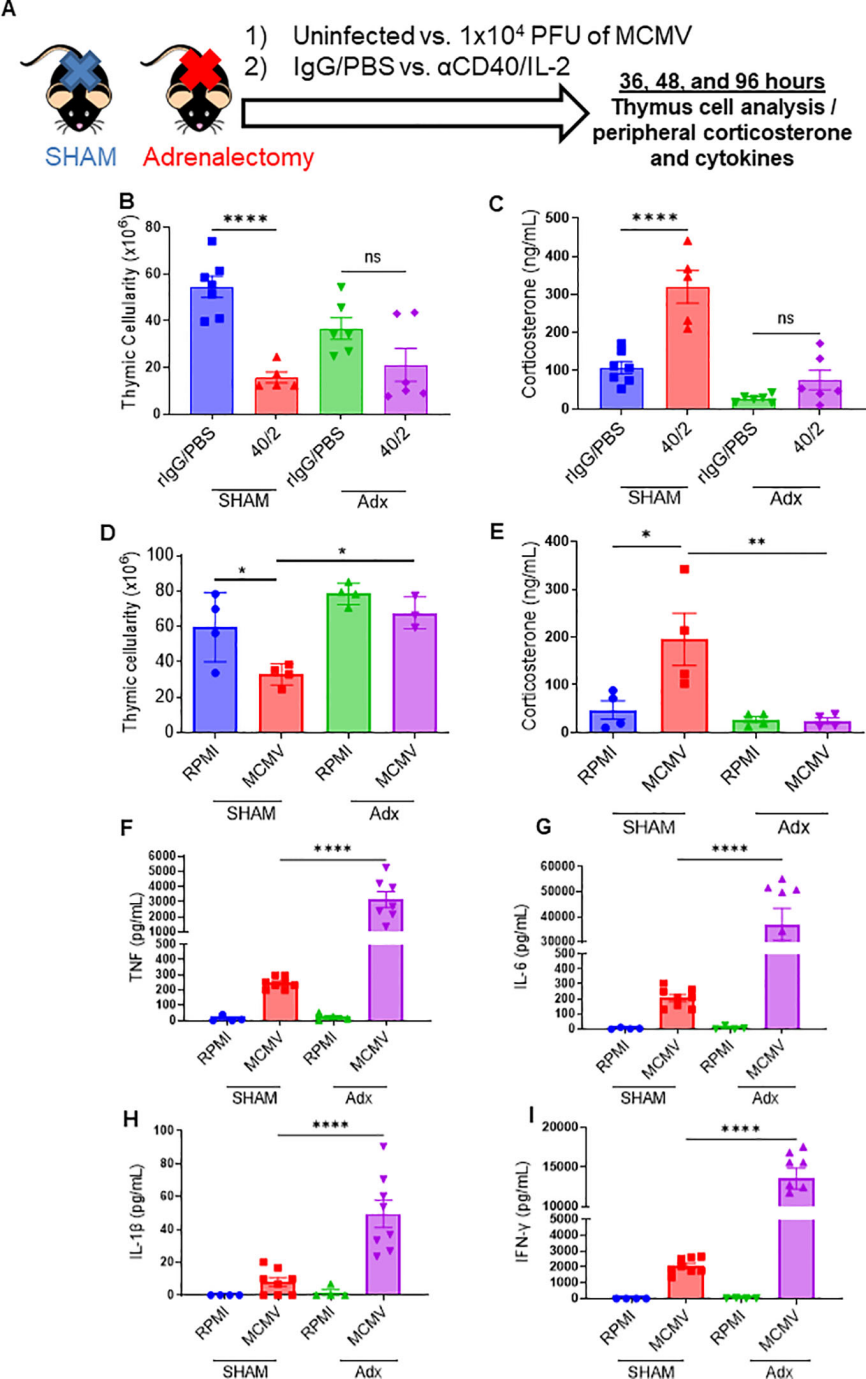
Abrogation of the corticosterone response via adrenalectomy can protect the thymus from immunotherapy or viral infection associated thymic involution. **(A)** Schema – C57BL/6 mice either had a SHAM or adrenalectomy (adx.) surgery performed several weeks prior to the experiment. Adx and SHAM mice were either inoculated with 1x10^4^ PFU of MCMV (controls received 0.2 mL of RPMI), or were injected with aCD40 (65μg) in combination with rhIL-2 (5x10^6^ IU) (controls received 0.2 mL of PBS). Mice were assessed at 36 hrs, DPI 2, and DPI 4. **(B, C)** Thymic cellularity and serum corticosterone of SHAM and adx mice, 2 days after aCD40/IL-2 treatment. **(D, E)** Thymic cellularity and serum corticosterone of SHAM and adx mice, 2 days after aCD40/IL-2 treatment 36 hours after MCMV inoculation. **(F–I)** Serum cytokine levels of TNF **(F)**, IL-6 **(G)**, IL-1β **(H)**, and IFN-y **(I)** 4 days after MCMV infection. **(B–I)** SEM bars, n=3-8 mice per group, representative of 2-3 combined experiments. One-way ANOVA with multiple comparisons based on means between groups was used to determine statistical significance; P<0.05*, P<0.01**, P<0.0001****. ns, non-significant.

Having determined that thymic involution occurred in the absence of proinflammatory cytokines during a systemic immune challenge, it was next investigated if systemic CS responses were responsible for the thymic involution after systemic immunotherapy. Mice were adrenalectomized to impair CS responses, as has previously been published (37, 38). SHAM and adrenalectomized (adx) mice were treated with anti-CD40/IL2 or challenged with primary subacute MCMV infection to assess effects on the thymus (**Figure 4A**). Following either regimen, adx mice demonstrated significant protection against thymic involution and cell loss when compared to SHAM mice (**Figures 4B, C**), which could be correlated with significantly diminished CS responses (**Figures 4D, E**). Adx mice also exhibited a markedly heightened pro-inflammatory cytokine profile following MCMV infection (**Figures 4F–I**), which could be predicted based on the well-characterized role of corticosteroids on down-modulating immune activation (39). While these higher cytokines did later cause increased morbidity and mortality in adx mice (data not shown), a short-term protective effect from thymic involution was still observed, implicating CS as the primary mediator of thymic involution as opposed to proinflammatory cytokines following systemic acute immune stimulation.

**Figure 5:**
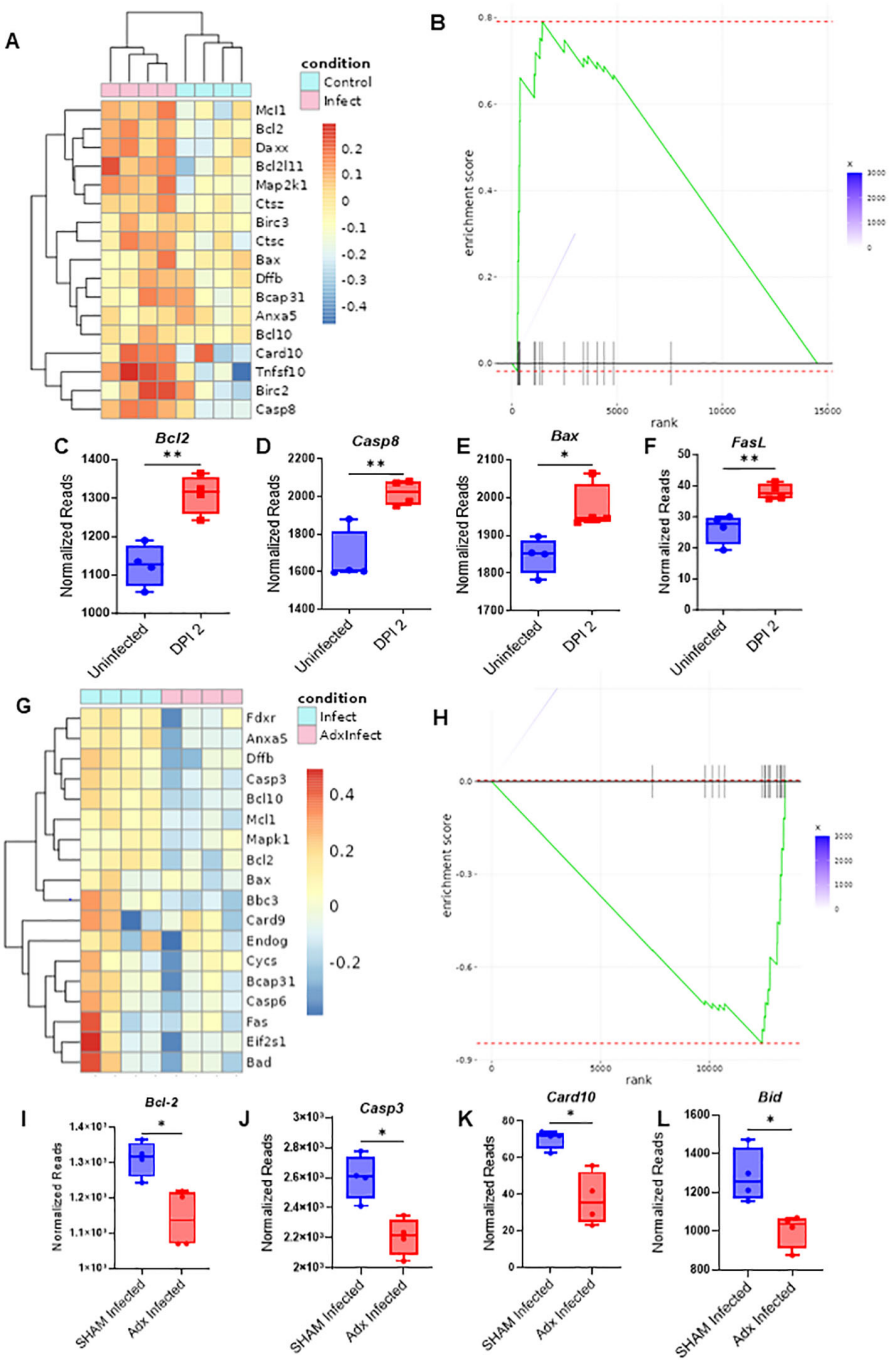
Adrenalectomized mice are protected from corticosterone mediated thymic apoptosis that occurs during an acute systemic immune challenge. **(A)** RNAseq heatmap of apoptosis related genes and their differential expression from the extracted RNA of thymocytes from SHAM control and SHAM MCMV infected (36 hours post infection) mice. **(B)** GSEA of apoptosis related genes in the heatmap of **(A)** with SHAM infected vs SHAM control as the basis of enrichment **(C–F)** Normalized reads of the genes *Bcl2* **(C)** *, Casp8* **(D)** *, Bax* **(E)** *, and FasL* **(F)** between SHAM control and SHAM infected mice 48 hours post infection. **(G)** Heatmap of apoptosis related gene differentials between SHAM infected and adx infected mice at 36 hours post infection. **(H)** GSEA of apoptosis related genes in the heatmap of 5G, with adx infected vs SHAM infected as the basis of enrichment. **(I–L)** Comparison of normalized reads of the genes *Bcl-2* **(I)** *, Casp3* **(J)** *, Card10* **(K)** *, and Bid* **(L)** between SHAM and adx infected mice. **(C–F, I–L)** SEM bars, n=4 mice per group. Student’s *t* test used to determine statistical significance; P<0.05*, P<0.01**.

To further distinguish the effects of CS vs pro-inflammatory cytokines in the context of thymic involution, the thymuses of SHAM and adx control and infected mice were examined through RNAseq analysis to determine differentials in the expression of apoptotic genes. Comparing control vs infected SHAM mice, RNAseq analysis revealed that 173 genes were differentially expressed (adjusted p<0.05) in infected mice in comparison to their control counterparts, with 166 genes noted as being upregulated, and only 7 as being downregulated (**Supplementary Figure 5A**). Key proapoptotic genes, such as *Casp8, Bax, and Fasl*, and others were at higher levels of expression in the thymocytes of infected mice at the 36-hour timepoint, coinciding with the peak of the CS response, while gene set enrichment analysis (GSEA) also revealed an upwards enrichment score of the assessed apoptosis related genes when comparing infected to control mice (**Figures 5A–F**; **Supplementary Figure 5B**). Gene Ontology (GO) term enrichment using the Kyoto Encyclopedia of Genes and Genomes (KEGG) and reactome databases indicated that terms associated with inflammation and apoptosis, such as “NOD-like pathway signaling,” “graft vs host disease,” “postsynaptic endocytosis,” “response to type 1 interferon,” and others, were differentially expressed between control and infected groups (**Supplementary Figures 5C, D**). These results confirm that acute viral infection causes marked increases in thymic apoptosis, which likely drives involution.

Infected SHAM and adx mice were next compared. RNAseq analysis revealed that a differential of 134 genes existed between the groups, with 106 genes being upregulated in infected SHAM mice when compared to adx, vs only 28 being downregulated (**Supplementary Figure 5E**). Infected SHAM mice displayed an elevated proapoptotic gene signature in comparison to their adx counterparts (**Figure 5G**; **Supplementary Figure 5F**). GSEA further supported this observation, revealing an enrichment of downregulated apoptosis genes when comparing adx infected mice to SHAM (**Figure 5H**). Key apoptotic genes, such as *Casp3, Card10, Bid*, and *Bcl-2*, were at a significantly higher level of expression in SHAM infected mice (**Figures 5I–K**), while other proapoptotic genes, like *Bax, Bad, Casp3*, and *Fas*, showed a trend of being higher (**Supplementary Figures 5G–J**), in addition to other differentially expressed apoptosis related genes as plotted via heatmap (**Figure 5G**; **Supplementary Figure 5F**). When adx infected mice were compared to SHAM infected via GO term enrichment, terms associated with apoptosis regulation showed a differential, such as “regulation of MDA-5 signaling” (which has been associated with apoptosis) and “regulation of apoptotic cell clearance” (**Supplementary Figure 5K**). To confirm that these effects were glucocorticoid related, the genes *Nr3c1*, well known as the encoder of the glucocorticoid receptor, and *Fkpb5*, which serves as a co-chaperone of the GC receptor, were analyzed. SHAM infected mice had much higher expression of these two genes, indicating the role being played by corticosterone, while adx mice demonstrated expression at or below the level of the control mice (**Supplementary Figures 5L, M**). Considering previous results demonstrated that adx mice are protected against thymic involution during an acute systemic challenge, these results indicated that CS acted as an inductor of apoptosis in the thymus, leading to acute thymic involution.

### Advanced aging impairs thymic recovery in mice infected with MCMV

**Figure 6:**
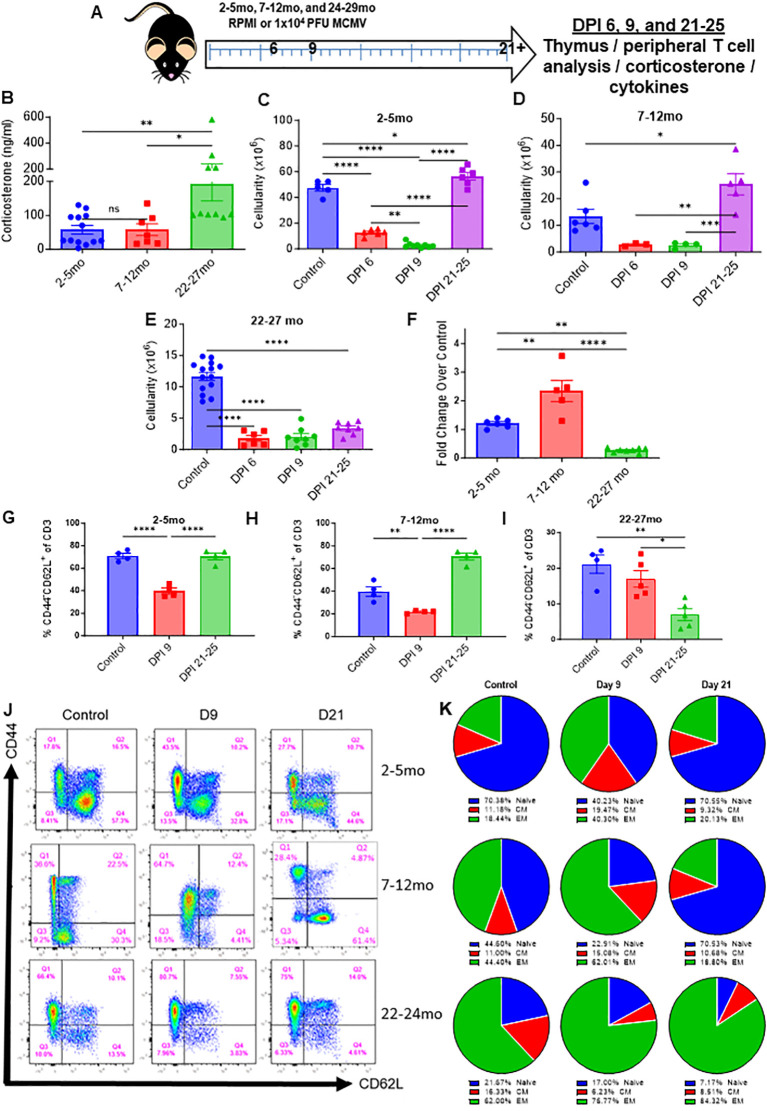
Age impairs thymic recovery and prolongs memory expansion in mice infected with MCMV. **(A)** Schema; young (2-5mo), middle aged (7-12mo), and aged (22-27mo) mice were infected with low dose MCMV (1x10^4^ PFU) and assessed at days 3 (blood draw only), 6, 9, and 21 and 25. **(B)** Serum corticosterone levels of young, middle aged, and aged infected mice at DPI 3. **(C–F)** Thymic cellularity of control and infected young, middle aged, and aged mice at DPI 6, 9, and 21 through 25. **(G–I)** Percentages of naïve (CD44^-^CD62L^+/-^) CD3 T cells in the spleens of young, middle aged, and aged mice controls and infected mice assessed at DPI 9 and 21-25 **(J)** Representative flow cytometry of the spleens of infected young, middle aged, and aged mice at days 9 and 21-25, as well as control mice for each age group. **(K)** Pie chart representation of the naïve, central memory, and effector memory ratios of young, middle aged, and aged control and infected mice at days 9 and 21-25. SEM bars, n=3-15 mice, representative of 2-4 experiments. One-way ANOVA with multiple comparisons based on means between groups was used to determine statistical significance in all panels; P<0.05*, P<0.01**, P<0.001***, P<0.0001****.

Profound thymic involution progressively and naturally occurs with age, markedly reducing the production of naïve T-cells and shifting the T-cell pool towards a memory dominant one. It has not been documented if age has an effect on thymic recovery and rebound, and because the elderly population face elevated pathology during viral infections and are more likely to develop cancer that would necessitate the usage of immunostimulatory therapy, it was next assessed what impact age would have on the reconstitution of the thymus following immune challenge. Young (2-5mo, approximately equivalent to a 20-30 year old human), middle aged (7-12mo, approximately equivalent to a 35-50 year old human), and advanced aged (22-24mo, approximately equivalent to a 60-70 year old human) were infected with low dose MCMV to determine differentials in stress response, thymic involution, and thymic recovery (**Figure 6A**). Corticosterone assessment at day 3 post infection (DPI) revealed that aged mice still had elevated corticosterone in comparison to their young and middle-aged counterparts, which was striking considering that earlier data showed that young mice had a transient peak of corticosterone at 36 hours during MCMV challenge, which had resolved by day 2 (**Figure 6B**; **Supplementary Figure 2D**). This could indicate that aged mice may have an amplified and/or prolonged corticosterone response. Thymic cellularity was demonstrably diminished in comparison to control mice at DPI 6 and 9 in all groups, but by days 21-25, young and middle-aged mice had experienced thymic rebound hyperplasia in a similar manner to what was previously shown with immunotherapy and immune challenge (**Figures 6C–E**). Aged mice, however, showed no signs of thymic recovery at this timepoint, and instead had thymic cellularity comparable to when peak infection was occurring at days 6 and 9 (**Figure 6E**). While middle aged and aged mice had comparably smaller thymuses at baseline in comparison to young mice, middle aged mice were still able to produce a thymic rebound effect following acute viral resolution. Interestingly, when considering thymic cellularity fold change in comparison to control mice, middle aged mice had a greater rebound effect than young mice, and some middle-aged mice had comparable thymic cellularities to young mice after this rebound, while the lack of recovery in the advanced aged mice was highlighted even further (**Figure 6F**). This could indicate that the increased levels of CS observed in aged mice at day 3, which was not present in young and middle-aged mice, may have further deleterious effects on thymic recovery coupled with possible thymic epithelial damage associated with increased aging.

Because aged mice already have a memory dominant T-cell pool at homeostasis, it was next investigated as to what effect infection would have on aged naïve and memory populations, considering it was observed earlier with immune therapy and challenge in young mice that infection produces marked memory T-cell expansion that eventually recedes. Aged mice at baseline had a substantially smaller percentage and number of peripheral naïve T cells and a larger percentage and number of memory CD3 T-cells in comparison to young and middle aged mice (**Figures 6G–K**; **Supplementary Figure 6A–D**). By DPI 9, where peak MCMV pathology can be observed, all age groups of mice had significant memory T-cell expansion and diminished naïve T-cell presence (**Figures 6G–K**; **Supplementary Figures 6A–D**). Middle aged mice proportionally had the largest increase in memory T-cells, while the already memory dominated aged T-cell pool became further skewed towards memory. By days 21-25, the time when the acute phase of MCMV infection has resolved, young and middle-aged mice saw a reconstitution of naïve T cells and a recession of memory, in line with what was previously observed with immune therapy and challenge. Aged mice, however, were even further skewed towards memory, even more so than during the peak of MCMV pathology, while having even less naïve T cells than at baseline (**Figures 6G–K**; **Supplementary Figures 6A–D**). The thymic rebound observed in young and middle-aged mice is likely the primary factor in the restoration of the naïve T cell population, while lack of this rebound in aged mice could explain why even less naïve T cells were present than at baseline. These results indicate that aged mice have an amplified and potentially elongated corticosterone response that results in marked thymic involution, in thymuses that have already naturally involuted with age. The lack of thymic rebound and naïve T cells post MCMV clearance could mean that these mice are more susceptible to novel antigens following immune challenge, which is a factor that could be critical in numerous clinical settings.

## Discussion

Stress responses are induced following various tissue insults, being primarily mediated by glucocorticoids as well as other factors like catecholamines and exert numerous functions in mediating tissue homeostasis by suppressing pro-inflammatory processes. These responses are critical in preventing or mitigating subsequent immunopathology and tissue destruction that could result from an unregulated immune response. Glucocorticoids have broad immunosuppressive effects that influence multiple immune cell-types, and are commonly used in a variety of clinical contexts, whether it be for the modulation of autoimmunity (40–42), acute inflammatory conditions arising from pathogens (43–45), or for controlling adverse immune responses after transplantation (46–49).

Immunotherapy has become prominent in cancer therapy regimens due to the significant successes attributed to it. Immunotherapy can be considered a broad label, with many different types of regimens/reagents/cells being used. Cancer immunotherapies usually focus on producing and/or amplifying an immune anti-tumor response, either through direct stimulatory regimens or the removal of inhibitory pathways/cells, as well as the use of direct immune cell transfers. These therapies are also being applied more in conjunction with conventional therapies. While the emphasis has centered on the effects of such therapies in peripheral immune responses or to the disease being treated itself (i.e., anti-tumor effects in the case of cancer), other components of immune cell biology and physiological reactions to the stimuli may also be impacted, such as stress responses.

From an evolutionary perspective, these immunosuppressive pathways may serve to control immune inflammatory pathways, protecting tissues from immunopathology caused by both the pathogen and the immune cells attacking the pathogen. In the context of cancer, however, these responses may be inhibitory to anti-tumor efficacy. The induction of such stress responses are dynamic, varied in intensity, and contingent on the extent of the perturbation, but often can be produced by strong systemic immunostimulation, such as that seen during acute viral infections or sepsis, as well as by systemic immunostimulatory therapies, like those used in cancer treatment. These conditions all activate innate immune cells such a macrophages, which can start a pro-inflammatory cytokine cascade meant to further amplify immune responses, but which can be difficult to control (33, 36, 50, 51). In certain circumstances, a “cytokine storm,” driven by such responses due to dysregulation of control mechanisms, can drive morbidity and mortality during acute viral infections and during immune therapy, necessitating the use of exogenous glucocorticoid administration and/or cytokine blockade to augment the induced stress response, as has been reported elsewhere (52, 53). Thus, stress responses are considered critical for immune cell control.

Outside of the direct effects on particular immune cell-types, certain immune organs, such as the thymus, which is the central organ for T-cell development, are also very sensitive to glucocorticoids effects. This is largely due to the extreme sensitivity of certain T-cell progenitors to apoptosis, given their stage in differentiation and the process of thymic education. In these cases, glucocorticoids induce apoptosis of thymocytes, and rapid thymic involution can occur. This is likely meant as a control pathway to circumvent any effects these pro-inflammatory processes may exert on thymic education and inappropriate selection.

The results presented here indicate that strong systemic immunostimulation, such as that used in cancer immunotherapy application or experienced during viral infections, can have profound effects on the thymus and T-progenitor cell development, potentially impacting the pool of naïve T cells in the periphery, as was observable both in mice and humans. It is not yet clear as to what the exact threshold of immunostimulation is that can induce this inhibitory effect, although the data presented here utilizing a subacute MCMV infection model indicates that induction of systemic pro-inflammatory cytokines, as observed with HD IL-2 immunotherapy, is not required. Only glucocorticoid induction appears pre-requisite for the thymic effects. Nonetheless, the induction of the proinflammatory cytokines with the other regimens does not rule out the possibility that they may contribute to the thymic involution, and they have been demonstrated to contribute to the induction of a glucocorticoid response as well (54). However, the observation that adx mice have protection against thymic involution during immune stimulation, despite having increased cytokine levels followed by mortality, strongly indicates that proinflammatory cytokines are not the primary driver of thymocyte apoptosis and involution.

The current study involved extremely strong systemic immunostimulation, which may not be reflective of other types/regimens of immunotherapy applied. For example, ICI targeting of PD-1/PD-L1 showed no significant effects of on the thymus. The results presented here also need to be taken in the context of immunostimulatory regimens involving only immunotherapy or infection, the latter shown only in mice. It remains to be determined if immunostimulation combined with other conventional cancer therapies (i.e., chemotherapy or radiation therapy) could further augment or amplify these effects. These conventional cancer therapies also likely contribute to stress responses, and therefore should be assessed for their capacity to potentiate the effects observed here. It should also be considered that cancer as a disease is widely accepted to be a significant source of psychological stress to an individual, notably having an impact on the neuroendocrine system which mediates stress hormone levels (55). This should also be investigated in the context of potential effects on the thymus and additive effects of therapeutic interventions.

Another important distinction between mice and humans is the maintenance of naïve T cells, with a report detailing that mice accomplish this maintenance through the thymus, while humans are more dependent on extended naïve T cell lifespan and proliferation in the periphery (56). However, the skewing of the T cell repertoire towards one that is memory dominant that occurs with age is well documented, as is thymic involution and the reduction of peripheral naïve T cells (57), so even though prolonged T cell lifespan is critical to human T cell maintenance, the importance of the thymus and its involution cannot be understated. It is thus likely that a combination of thymic output and peripheral T cell maintenance that sustains the naïve T cell population in humans. While the *in vivo* data here may not be fully comparable to the human condition, insight into how glucocorticoids effect the thymus and peripheral T cell pool is still valuable, with parallels being observed between data generated *in vivo* and with humans receiving IT. Future studies will likely be able to bridge the gap between the stress mediated effects on the thymus and the effects on peripheral T cell maintenance.

Thymic involution naturally occurs with age, which has been noted to impair thymic functionality, naïve T cell output, and to cause a memory T cell dominant phenotype. The elderly population is at an increased risk of developing cancer or having severe pathology from pathogens like viruses and are thus the more likely to receive IT as opposed to a young individual. The data presented in this manuscript indicates that the aged thymus is still vulnerable to further involution during acute stressors, such as viral infection, which further diminishes the presence of naïve T cells, while inflating the memory population further. Unlike younger animals, however, thymic rebound hyperplasia was notably impaired and not observed during these studies. This could be a consequence of impaired thymopoiesis following immune challenge, and this deficit could be deleterious to antigen priming. Notably, while young and middle-aged animals saw hyperplasia and a complete restoration of naïve T cells post infection, with higher numbers of naïve T cells in comparison to control mice, aged animals had even further skewing towards memory, and no evidence of naïve T cell restoration, even in comparison to aged controls that already had significantly diminished naïve T cell presence in comparison to younger animals.

It should be noted that while the studies presented here did not see thymic recovery or rebound in aged animals, this does not necessarily mean that rebound or recovery would not occur. It is possible that recovery of the thymus and naïve T cell population is delayed and would have been observed at a later timepoint. The prolonged CS response in aged mice that was observable at day 3 post-infection, at which point CS levels had subsided in younger animals, could also potentiate a delay in thymic reconstitution. Aged animals are also more susceptible to viral pathology, which could be a factor in the prolonged CS response. Another factor that should be considered is immune mediated pathology that occurs in aged animals, which is minimalized in younger animals. The data presented here using adrenalectomized mice, however, which have a substantially larger inflammatory response due to the lack of inhibition by glucocorticoids, would suggest that the inflammatory cytokines driving this immune pathology may not be contributors to thymic involution. However, the amplified presence of these inflammatory cytokines that is associated with the aged immune response may promote CS production as a compensatory mechanism, which could result in prolonged thymic atrophy and naïve T cell contraction. While these are all factors for consideration in the aged immune response, the data in this manuscript demonstrating a lack of thymic reconstitution, even if delayed instead of absent completely, is a phenomenon that warrants further mechanical dissection, as this prolonged impairment of thymopoiesis could be deleterious to antigen priming and primary T cell responses. It could also be of clinical relevance to investigate restoring this impaired thymopoiesis in aged individuals, which could be beneficial at a baseline in the context of novel antigen encounters, as well as after immune insult for similar reasons. These could both be investigated in the context of therapeutics like metyrapone, a GC inhibitor, which could alleviate these effects while maintaining adequate anti-tumor effects, while also allowing for evaluation of the mechanisms underlying GC mediated involution. A lack of mechanistic dissection does present a limitation in the studies performed in this manuscript, which future studies should focus on when interpreting the results that have been presented here.

It will be of interest to assess other critical variables, such sex and body mass index, with considerations to both stress responses and thymic/naïve T-cell alteration, as they are likely to affect thymic inhibitory responses in the context of immune therapy or acute viral infections. Obesity has been shown to accelerate thymic aging, which could have similar consequences to what has been shown with the aged data presented in this paper (58, 59). Furthermore, glucocorticoids are associated with the accumulation of adipose tissue, and obesity itself is associated with elevated levels of glucocorticoids, which could alter thymic function at a baseline and during an immune response (60). In the context of immune stimulation, this may have more meaningful consequences on the ability to mount later primary T- cell responses, while also potentially impairing primary T cell responses during immune challenges. It should be noted that aged animals typically have higher amounts of adipose tissue, which could be a factor in acute thymic involution.

The effects of sex hormones, such as estrogen and testosterone, have also been shown to augment thymic aging associated involution *in vivo*, with data indicating that estrogen may play a role in thymic regulation and involution, though this hasn’t been extensively characterized (61). Sex hormones and androgens have also been demonstrated to be closely linked with glucocorticoid production, with oestrogen administration in males having being shown to induce a cortisol stress response (62). Estrogen has also been shown to be inhibitory to thymic proliferation and a promotor of thymic apoptosis as well, which is of note given the data generated in this manuscript was done so with female mice (63). Obesity in males is associated with higher levels of estrogen at a baseline, which could play a role in thymic involution and the ability to mount a rebound, though this too has not been very well characterized (64). This should also be considered in the context of aging, which brings substantial changes in adipose tissue accumulation and sex hormone regulation, which highlights the complexity and interconnectivity of these factors. The body of publications investigating the effects of age, obesity, and sex on thymic rebound are sparse, despite these being critical factors in the context of clinical evaluation and treatment. The data presented in this manuscript would warrant investigation into these factors, as they may also have an inhibitory effect on thymic rebound and naive T cell reconstitution following an acute immune response. It is also worth noting that the human data presented in this manuscript did not factor in patient sex, a limitation which should be taken into consideration when drawing conclusions.

The *in vivo* data presented here suggest a direct effect of glucocorticoid responses on thymic involution, responses that may be given the protection observed in adx mice against immunostimulatory-induced involution. However, although adx mice were protected from initial thymic involution despite increased pro-inflammatory cytokine responses, the mortality that resulted during the studies highlights the need for glucocorticoid-mediated immunosuppression. The well-characterized role of adrenal function in tissue homeostasis and the general maintenance of health means that a cost to benefit ratio needs to be determined when attempting to inhibit adrenal function to achieve thymic protective effects (54, 65). It is also worth considering that adrenalectomy not only effects the production of glucocorticoids, but also other such factors and hormones, such as mineralocorticoids and catecholamines, which could also have an effect on the immune response. This stands as a current limitation of modeling, however, as, the usage of a cre system to eliminate endogenous glucocorticoids would be ideal, but such a model does not currently exist.

These results demonstrate that the inhibitory effects of systemic immunotherapy can influence the thymus, but the effects are relatively transient in nature in young mice, while being comparatively prolonged in aged mice. However, many mouse preclinical tumor immunotherapy studies utilize short-term treatment regimens, which can be at variance with clinical cancer immunotherapy, in which longer time-courses are applied. Thus, the many variables (genetically homogeneous mice/heterogeneous people; young mice/older tumor-bearing people; short vs long treatment course, and other features) make extrapolation of these findings to clinical practice impractical. Much more information is required to exploit these findings for patient benefit. There may cumulatively be more pronounced effects or damage on the thymus that could occur with repeated or prolonged immunotherapy, affecting thymic recovery and naïve T cell production. The results presented here indicate that assessing the effects of immunotherapy regimens on stress and its subsequent immunologic effects may reveal biology that could favorably alter host-tumor interactions to boost host defense. At least some questions emerge from the work that could guide further inquiry.

## Data Availability

The datasets presented in this study can be found in online repositories. The names of the repository/repositories and accession number(s) can be found below: PRJNA1117947 (SRA).

## Supplementary material

Supplementary Figure 1 Strong systemic immunostimulatory regimens cause memory CD8 T cell expansion, while immune checkpoint inhibition does not produce thymic events. **(A, B)** Populations of double negative, single, and double positive thymocytes over the course of the 33 day experiment, shown in percents **(A)** and total thymocyte counts **(B)**. **(C, D)** Breakdown of the double negative subsets by percentage **(C)** and total thymocyte counts **(D)**. DN1: CD44^+^, CD25^-^. DN2: CD44^+^, CD25^-^. DN3: CD44^-^, CD25^+^. DN4: CD44^-^, CD25^-^. S1E: Naïve and memory percentages of CD8 (top) and CD4 (bottom) T cell subsets over the course of the experiments described in figure 1’s schema (**Figure 1A**).

Supplementary Figure 2 Mice with sub-acute primary MCMV infection also present with thymic involution and increased corticosterone levels but not increased pro-inflammatory cytokines. **(A)** Schema – C57BL/6 mice were infected with 1x10^4^ PFU MCMV, (sub-acute infection), while control mice received 0.2mL of RPMI. Mice were assessed at 36hrs, DPI 2 (days post injection), and DPI 4 and assessed for thymus phenotype and systemic readouts. **(B)** Thymic cellularity of control and infected mice at DPI 4. **(C)** Serum corticosterone levels of mice 36 hours post inoculation and at DPI 2. **(D–G)** Percentages of memory vs naïve CD4^+^ **(D, F)** and CD8^+^ **(E, G)** of spleens assessed at DPI 4. S3H: Thymic cellularity of mice at 36 hours post infection. **(I, J)** Serum cytokine levels of TNF and IL-6 from control and infected mice at DPI 4. **(K, L)** Relative gene expression of *IL-*6Ra and *TNFRSF1A* from spleens of control and infected mice assessed at DPI 4. **(B, C, D–G, H, I–L)** n= 3-8 mice per group, representative of 1-2 combined experiments. Student’s *t* test used to determine statistical significance; P<0.05*, P<0.01**, P<0.001***. **(C)** SEM bars, n=4-8 mice, representative of two experiments. One-way ANOVA with multiple comparisons based on means between groups was used to determine statistical significance; P<0.01**, P<0.001***.

Supplementary Figure 3 HD IL-2 treatment causes apoptosis driven thymic involution mediated by corticosterone **(A)** Representative flow cytometry plots (CD4 vs CD8) of PBS and IL-2 treated mice at days 2, 12, and 33. **(B)** Thymic cellularity of mice treated with either PBS or HD IL-2 at day 2. **(C)** Percent of annexin V^+^ CD3 T cells 24 hours after receiving IL-2. **(D)** Corticosterone fold change of IL-2 treated vs untreated mice at days 2, 12, and 19. **(E, F)** Populations of double negative, single, and double positive thymocytes over the course of the 33 day experiment, shown in percents **(E)** and total thymocyte counts **(F)**. **(G, H)** Fold change of naïve, effector memory, and central memory CD8 **(G)** and CD4 **(H)** T cells in the spleens of mice assessed over the course of the 33 day experiment. **(B, C)** SEM bars, n=4-8 mice per group, representative of 2 experiments. Student’s T test used to determine statistical significance; P<0.05*, p<0.01**. **(G, H)** SEM bars, n=3-4 mice, representative of 1-2 experiments. One-way ANOVA with multiple comparisons based on means between groups was used to determine statistical significance; P<0.05*.

Supplementary Figure 4 Immune therapy causes naive T cell loss and memory T cell expansion. **(A)**: Representative flow plots showing CD4 and CD8 gating on CD3, as well as representative plots of CD45RA^+^ vs CD45RO^+^ at baseline and day 8 after receiving IL-2 (**B)** Representative flow plots showing CD45RA and CD45RO staining on CD3s at baseline, day 2, and day 8. **(C)** HLA-DR quantification of CD3^+^ cells at baseline, day 2, and day8. **(D–F)** CD4 **(D)**, CD8 **(E)** and total **(F)** TREC quantification of human clinical studies at baseline, day 8 (CD4 and CD8 only), and week 4 (total only). (**C)** n=4 patients, representative of the samples from one of the clinical trials. One-way ANOVA with multiple comparisons based on means between groups was used to determine statistical significance; P<0.05*, P<0.01**. (**D, E)** n=9 samples, representative of two clinical trials. Paired *t* test was used to determine statistical significance.

Supplementary Figure 5 Adx mice have higher levels of serum cytokines due to a lack of corticosterone modulation, but the thymus is still protected from apoptosis despite these elevated levels. S5A:. Volcano plot showing differential expressed, up regulated, and down regulated genes of SHAM control vs infected mice at 36 hours post inoculation. S5B: Continued gene map of differentially expressed apoptotic genes between SHAM control and SHAM infected mice at 36 hours, showing genes with larger differentials in expression. S5C and S5D: Go term enrichment analysis of the KEGG and reactosome pathways when comparing SHAM infected vs SHAM control mice. S5E: Volcano plot showing differential expressed, up regulated, and down regulated genes of adx vs SHAM infected mice at 36 hours post inoculation. S5F: Continued gene map of differentially expressed apoptotic genes between SHAM infected and adx infected mice at 36 hours, showing genes with larger differentials in expression. S5G – S5J: Normalized reads of *Bax* (S5G)*, Bad* (S5H)*, Casp3* (S5I)*, and Fas* (S5J) in the thymuses of infected SHAM vs adx mice 36 hours post inoculation. S5K: Go term analysis of reactosome and general biological pathways of adx vs control mice at 36 hours post infection. S5L and S5M: Normalized reads of *Nr3c1* and *Fkbp5* in the thymuses of control, infected SHAM, and infected adx mice 36 hours post inoculation S5G – S5H: SEM bars, n=4 mice per group, representative of 1 experiment. Student’s *t* test used to determine that statistical differences did not exist between SHAM infected and adx infected groups. S5L – S5M: SEM bars, n=4 mice per group, representative of 1 experiment. One-way ANOVA with multiple comparisons based on means between groups was used to determine statistical significance; P<0.05*.

Supplementary Figure 6 Contraction of peripheral naïve and expansion of memory T cells is prolonged in aged mice. **(A, B)** Percent quantification of central memory and effector memory CD3^+^ subsets in young, middle aged, and aged mice over the course of 21-25 days. **(C, D)** Cell numbers quantification of central memory and effector memory CD3^+^ subsets in young, middle aged, and aged mice over the course of 21-25 days. n= 4-5 mice per timepoint, SEM bars. One way ANOVA was used to examine all graphs. P<0.05*, P<0.01**, P<0.001***, P<0.0001****.
