# Supplementary figures and images for: Systemic immunostimulation induces glucocorticoid-mediated thymic involution succeeded by rebound hyperplasia which is impaired in aged recipients
Source: Front Immunol. 2024 Sep 9;15:1429912. doi: 10.3389/fimmu.2024.1429912 (PMC11416920; doi:10.3389/fimmu.2024.1429912)

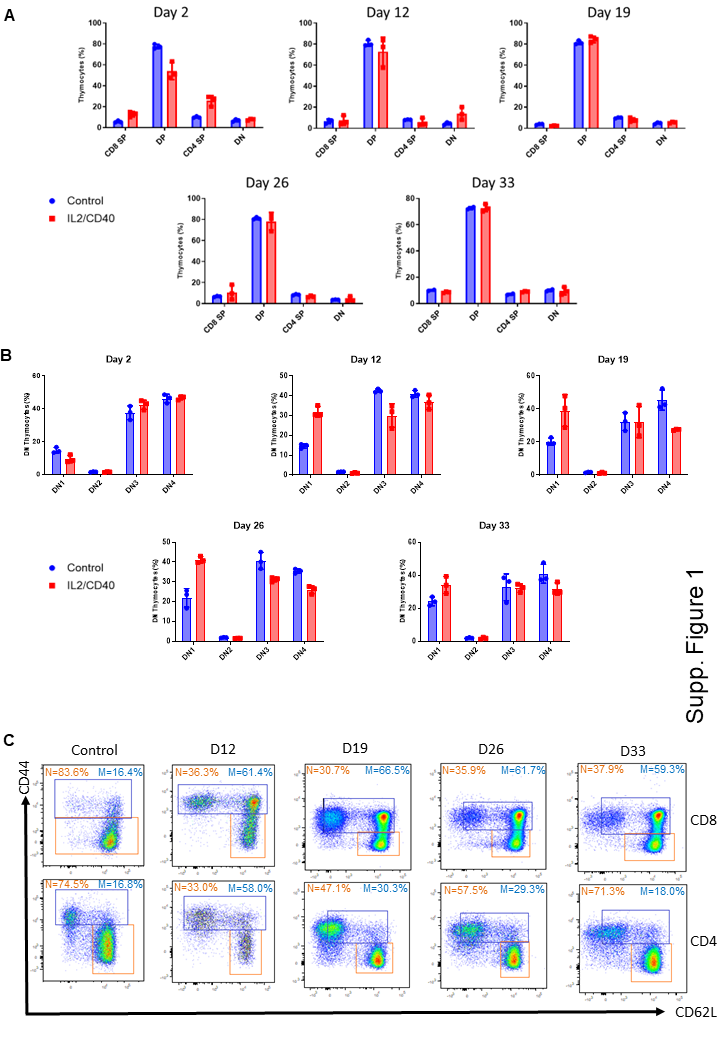

Supplement: Supplementary Figure 1 — Strong systemic immunostimulatory regimens cause memory CD8 T cell expansion, while immune checkpoint inhibition does not produce thymic events. (A, B) Populations of double negative, single, and double positive thymocytes over the course of the 33 day experiment, shown in percents (A) and total thymocyte counts (B). (C, D) Breakdown of the double negative subsets by percentage (C) and total thymocyte counts (D). DN1: CD44+, CD25-. DN2: CD44+, CD25-. DN3: CD44-, CD25+. DN4: CD44-, CD25-. S1E: Naïve and memory percentages of CD8 (top) and CD4 (bottom) T cell subsets over the course of the experiments described in figure 1’s schema ( Figure 1A ). [file Image1.tif]

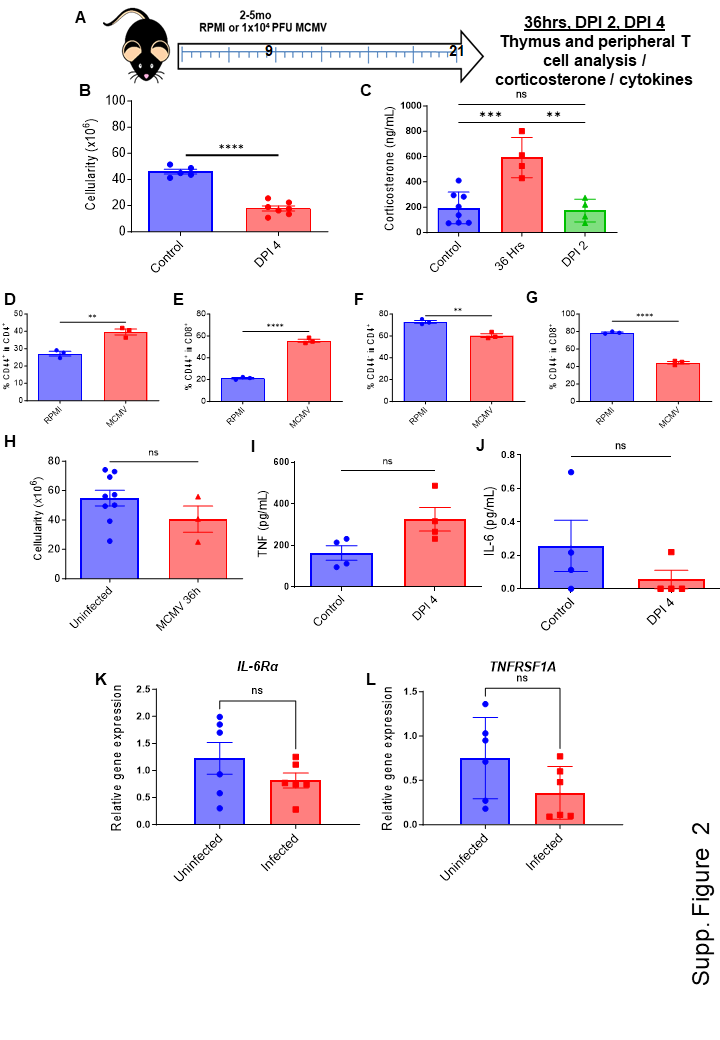

Supplement: Supplementary Figure 2 — Mice with sub-acute primary MCMV infection also present with thymic involution and increased corticosterone levels but not increased pro-inflammatory cytokines. (A) Schema – C57BL/6 mice were infected with 1x10^4^ PFU MCMV, (sub-acute infection), while control mice received 0.2mL of RPMI. Mice were assessed at 36hrs, DPI 2 (days post injection), and DPI 4 and assessed for thymus phenotype and systemic readouts. (B) Thymic cellularity of control and infected mice at DPI 4. (C) Serum corticosterone levels of mice 36 hours post inoculation and at DPI 2. (D–G) Percentages of memory vs naïve CD4+ (D, F) and CD8+ (E, G) of spleens assessed at DPI 4. S3H: Thymic cellularity of mice at 36 hours post infection. (I, J) Serum cytokine levels of TNF and IL-6 from control and infected mice at DPI 4. (K, L) Relative gene expression of IL-6Ra and TNFRSF1A from spleens of control and infected mice assessed at DPI 4. (B, C, D–G, H, I–L) n= 3-8 mice per group, representative of 1-2 combined experiments. Student’s t test used to determine statistical significance; P<0.05*, P<0.01**, P<0.001***. (C) SEM bars, n=4-8 mice, representative of two experiments. One-way ANOVA with multiple comparisons based on means between groups was used to determine statistical significance; P<0.01**, P<0.001***. [file Image2.tif]

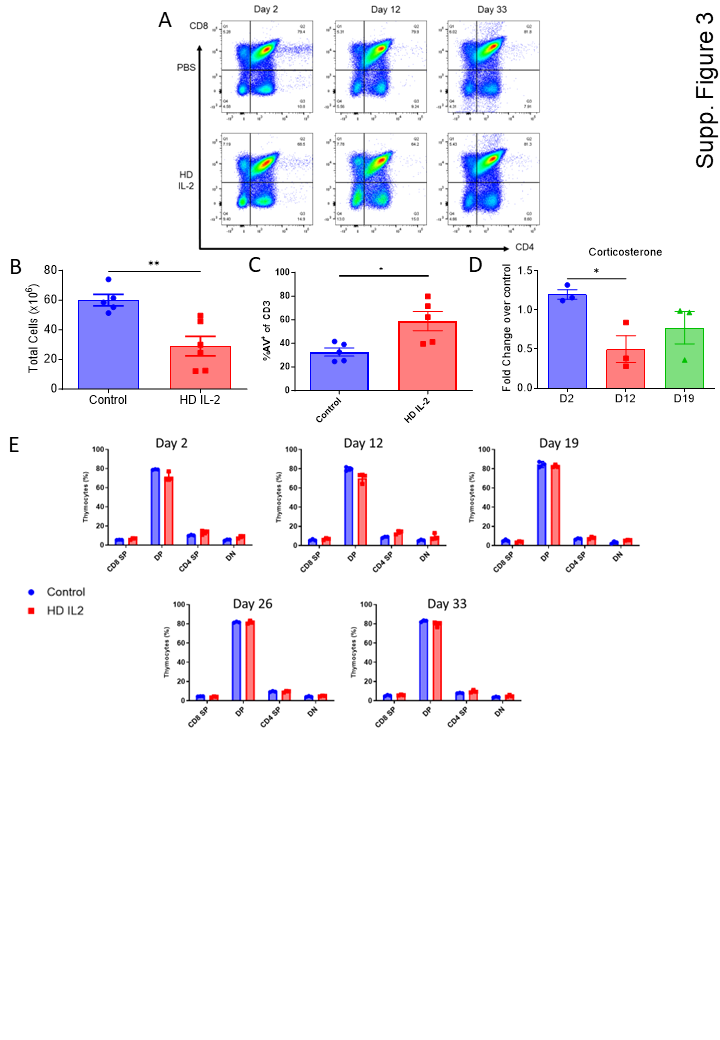

Supplement: Supplementary Figure 3 — HD IL-2 treatment causes apoptosis driven thymic involution mediated by corticosterone (A) Representative flow cytometry plots (CD4 vs CD8) of PBS and IL-2 treated mice at days 2, 12, and 33. (B) Thymic cellularity of mice treated with either PBS or HD IL-2 at day 2. (C) Percent of annexin V+ CD3 T cells 24 hours after receiving IL-2. (D) Corticosterone fold change of IL-2 treated vs untreated mice at days 2, 12, and 19. (E, F) Populations of double negative, single, and double positive thymocytes over the course of the 33 day experiment, shown in percents (E) and total thymocyte counts (F). (G, H) Fold change of naïve, effector memory, and central memory CD8 (G) and CD4 (H) T cells in the spleens of mice assessed over the course of the 33 day experiment. (B, C) SEM bars, n=4-8 mice per group, representative of 2 experiments. Student’s T test used to determine statistical significance; P<0.05*, p<0.01**. (G, H) SEM bars, n=3-4 mice, representative of 1-2 experiments. One-way ANOVA with multiple comparisons based on means between groups was used to determine statistical significance; P<0.05*. [file Image3.tif]

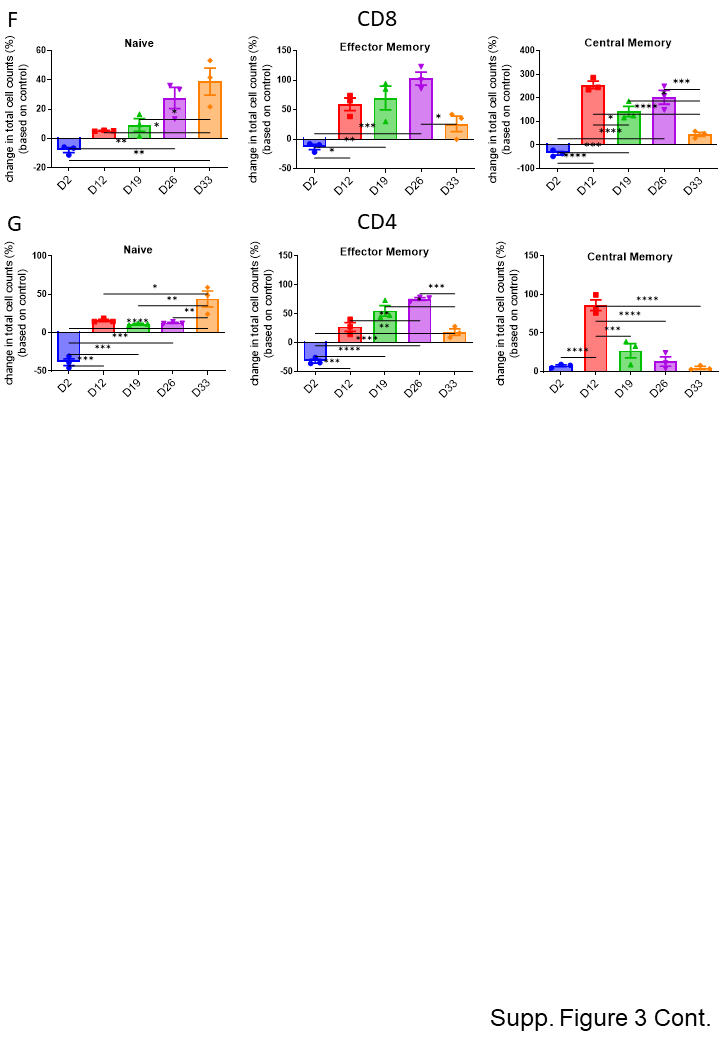

Supplement: Supplementary Figure 4 — Immune therapy causes naive T cell loss and memory T cell expansion. (A): Representative flow plots showing CD4 and CD8 gating on CD3, as well as representative plots of CD45RA+ vs CD45RO+ at baseline and day 8 after receiving IL-2 (B) Representative flow plots showing CD45RA and CD45RO staining on CD3s at baseline, day 2, and day 8. (C) HLA-DR quantification of CD3+ cells at baseline, day 2, and day8. (D–F) CD4 (D), CD8 (E) and total (F) TREC quantification of human clinical studies at baseline, day 8 (CD4 and CD8 only), and week 4 (total only). (C) n=4 patients, representative of the samples from one of the clinical trials. One-way ANOVA with multiple comparisons based on means between groups was used to determine statistical significance; P<0.05*, P<0.01**. (D, E) n=9 samples, representative of two clinical trials. Paired t test was used to determine statistical significance. [file Image4.tif]

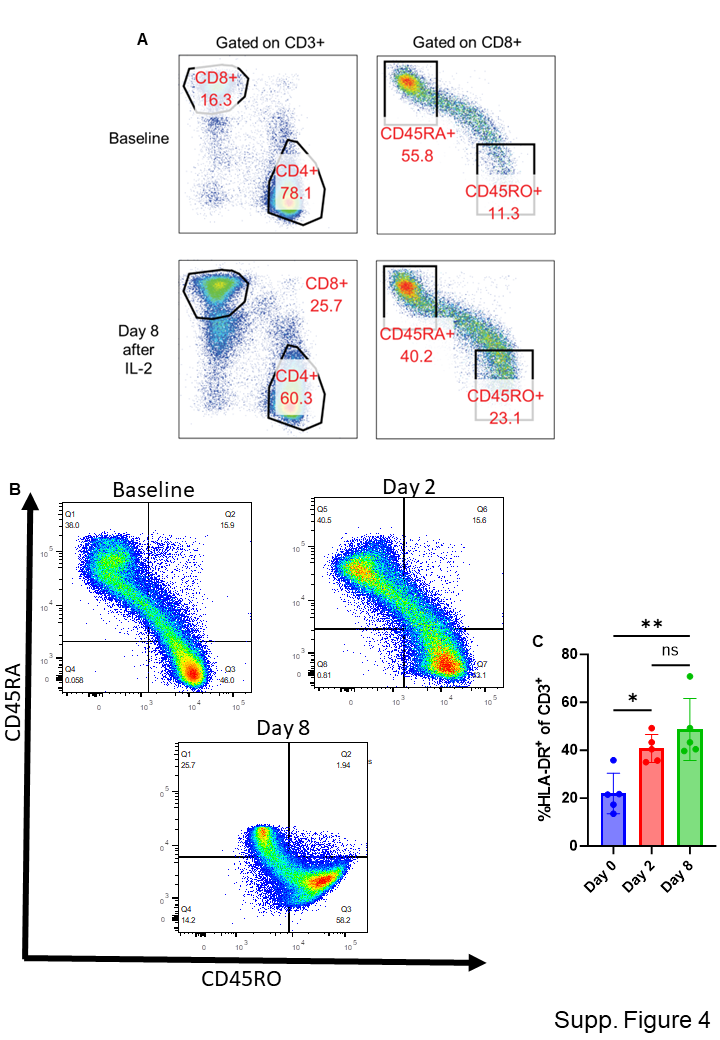

Supplement: Supplementary Figure 5 — Adx mice have higher levels of serum cytokines due to a lack of corticosterone modulation, but the thymus is still protected from apoptosis despite these elevated levels. S5A:. Volcano plot showing differential expressed, up regulated, and down regulated genes of SHAM control vs infected mice at 36 hours post inoculation. S5B: Continued gene map of differentially expressed apoptotic genes between SHAM control and SHAM infected mice at 36 hours, showing genes with larger differentials in expression. S5C and S5D: Go term enrichment analysis of the KEGG and reactosome pathways when comparing SHAM infected vs SHAM control mice. S5E: Volcano plot showing differential expressed, up regulated, and down regulated genes of adx vs SHAM infected mice at 36 hours post inoculation. S5F: Continued gene map of differentially expressed apoptotic genes between SHAM infected and adx infected mice at 36 hours, showing genes with larger differentials in expression. S5G – S5J: Normalized reads of Bax (S5G), Bad (S5H), Casp3 (S5I), and Fas (S5J) in the thymuses of infected SHAM vs adx mice 36 hours post inoculation. S5K: Go term analysis of reactosome and general biological pathways of adx vs control mice at 36 hours post infection. S5L and S5M: Normalized reads of Nr3c1 and Fkbp5 in the thymuses of control, infected SHAM, and infected adx mice 36 hours post inoculation S5G – S5H: SEM bars, n=4 mice per group, representative of 1 experiment. Student’s t test used to determine that statistical differences did not exist between SHAM infected and adx infected groups. S5L – S5M: SEM bars, n=4 mice per group, representative of 1 experiment. One-way ANOVA with multiple comparisons based on means between groups was used to determine statistical significance; P<0.05*. [file Image5.tif]

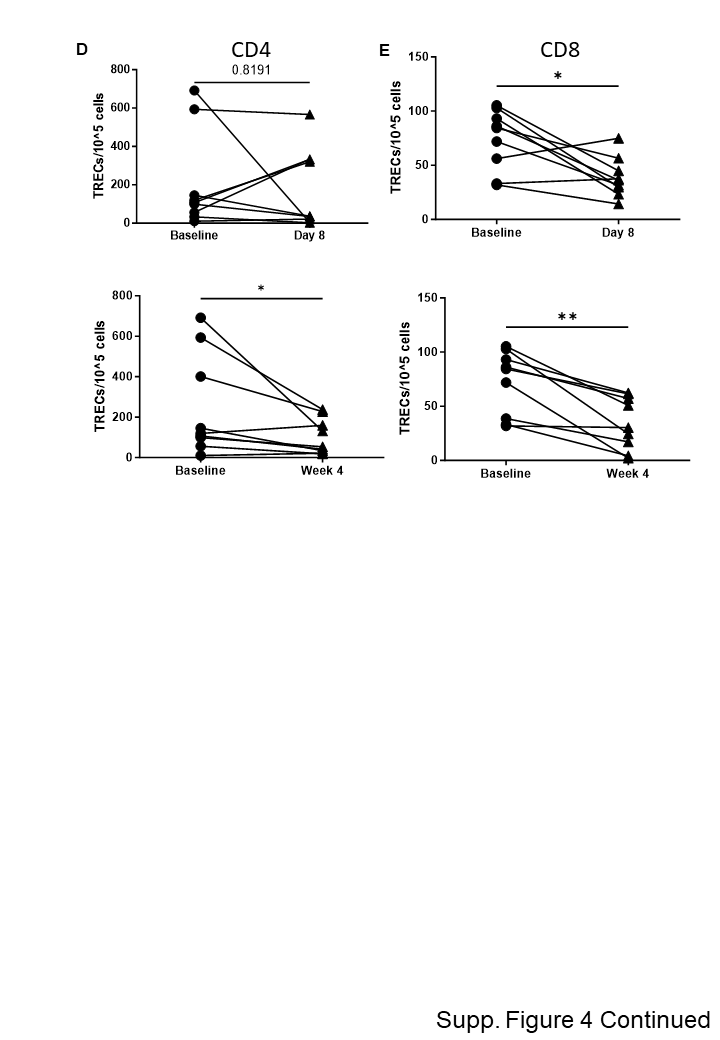

Supplement: Supplementary Figure 6 — Contraction of peripheral naïve and expansion of memory T cells is prolonged in aged mice. (A, B) Percent quantification of central memory and effector memory CD3+ subsets in young, middle aged, and aged mice over the course of 21-25 days. (C, D) Cell numbers quantification of central memory and effector memory CD3+ subsets in young, middle aged, and aged mice over the course of 21-25 days. n= 4-5 mice per timepoint, SEM bars. One way ANOVA was used to examine all graphs. P<0.05*, P<0.01**, P<0.001***, P<0.0001****. [file Image6.tif]

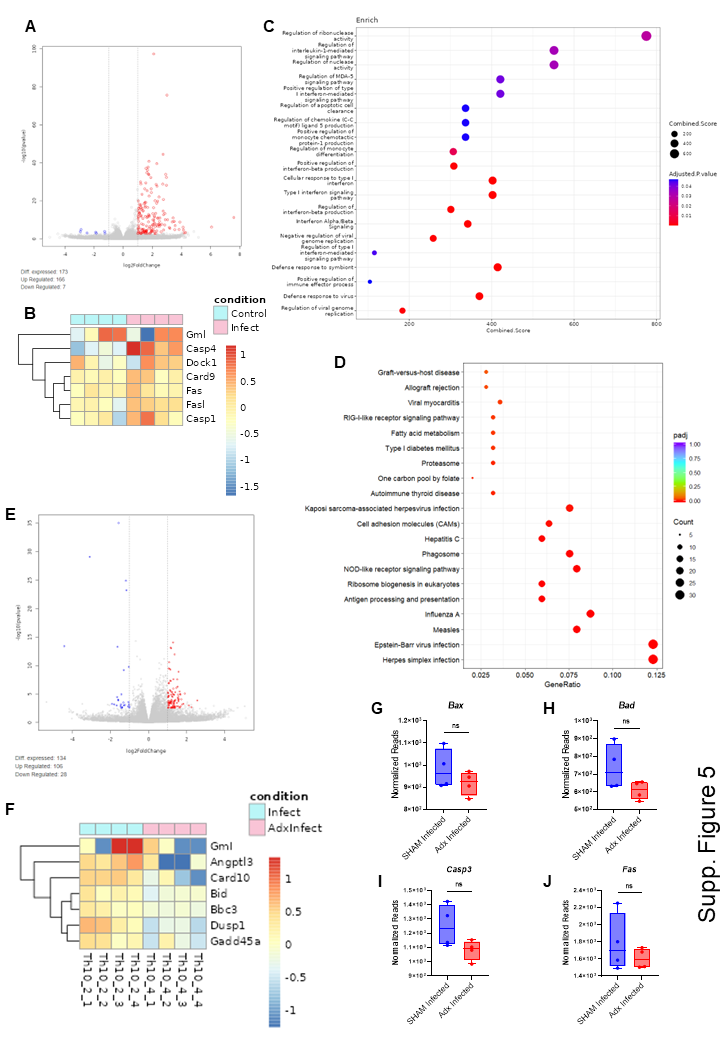

Supplement: Supplementary file 7 [file Image7.tif]

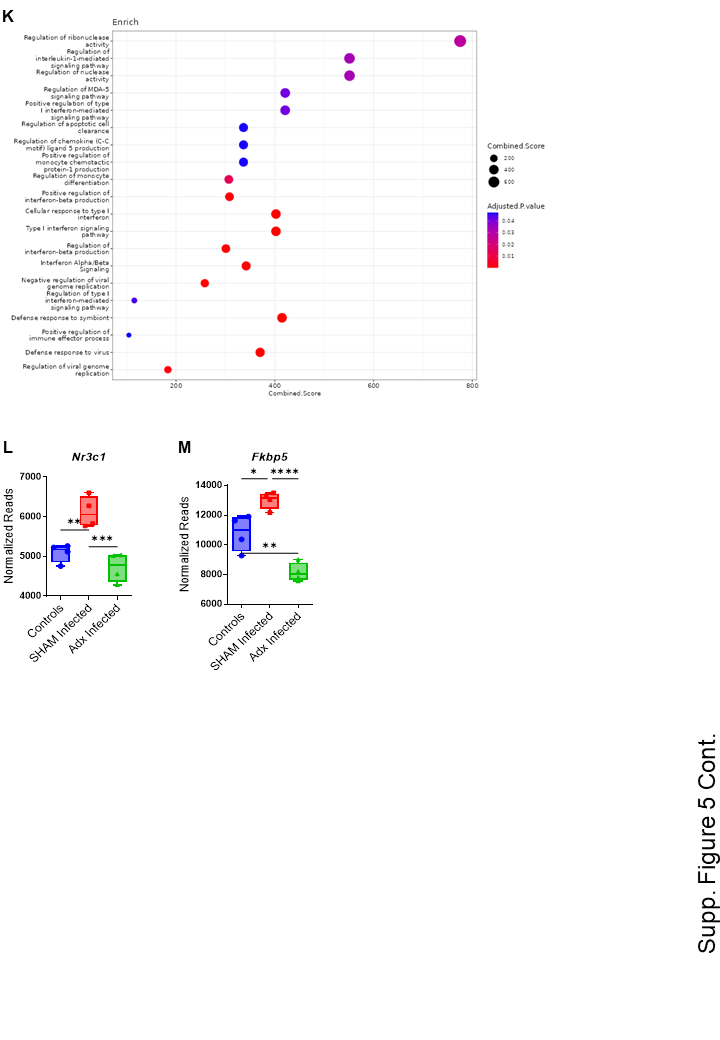

Supplement: Supplementary file 8 [file Image8.tif]

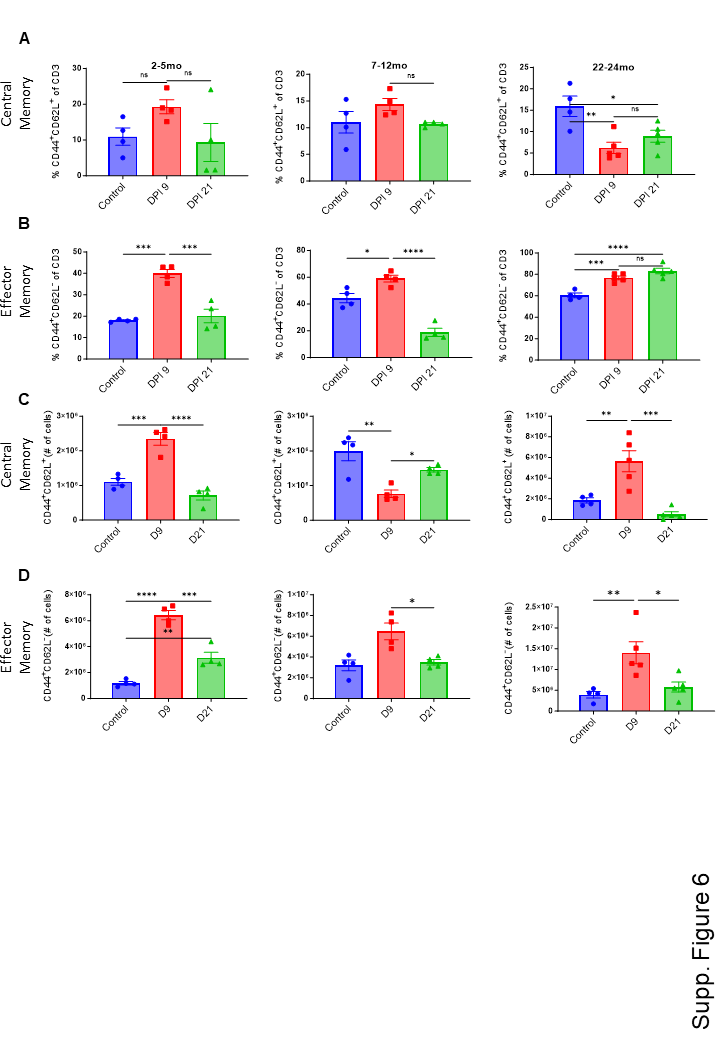

Supplement: Supplementary file 9 [file Image9.tif]
